# Group IVA Cytosolic Phospholipase A_2_ (cPLA_2_α): Pharmacological Inhibition Attenuates Kidney Inflammation and Fibrosis

**DOI:** 10.3390/biom16101430

**Published:** 2026-10-02

**Authors:** Berit Johansen, Said Movahedi Naini, Linn-Karina Selvik, Astrid Jullumstrø Feuerherm, Felicity J. Ashcroft, Chang Wang, Samuel Mon-Wei Yu, Wenqing Yin, Konstantinos Alevizopoulos, Oswald Quehenberger, Edward A. Dennis, Joseph V. Bonventre

**Affiliations:** 1 Department of Biology, Norwegian University of Science and Technology, N-7491 Trondheim, Norway; linn-karina.m.selvik@ntnu.no (L.-K.S.); astfe@tkmidt.no (A.J.F.); felicity.ashcroft@ntnu.no (F.J.A.); kalevizo@ventac-partners.com (K.A.); 2 Renal Division, Department of Medicine, Brigham and Women’s Hospital, Harvard Medical School, Boston, MA 02115, USAyinwenqing@gmail.com (W.Y.); 3 Coegin Pharma AB, c/o Medicon Village, Scheeletorget 1, 223 81 Lund, Sweden; 4 Department of Pharmacology, School of Medicine, University of California-San Diego, La Jolla, CA 90095, USAedennis@ucsd.edu (E.A.D.); 5 Harvard Stem Cell Institute, Cambridge, MA 02138, USA; 6 Division of Health Sciences and Technology, Harvard-Massachusetts Institute of Technology, Cambridge, MA 02139, USA

**Keywords:** chronic kidney disease, fibrosis, inflammation, lipid signaling

## Abstract

Chronic kidney disease (CKD) is a significant global health burden and a leading cause of death in the United States. Chronic inflammation contributes to fibrosis and CKD progression. Here, we present evidence that a key pro-inflammatory group IVA phospholipase A_2_ enzyme (cPLA_2_α) is critical to the development of kidney inflammation and fibrosis and can be targeted pharmacologically to attenuate CKD in animal models. Consistent with a role in human disease, overexpression of cPLA_2_α is seen in kidneys from patients suffering from CKD, and its expression is inversely correlated with the glomerular filtration rate. Genetic ablation of cPLA_2_α or inhibition with 1-octadeca-3,6,9,12,15-pentaenylsulfanyl-propan-2-one (AVX002) reduced inflammation and ameliorated renal fibrosis in mouse and rat disease models. In addition, reduced levels of eleven arachidonic acid-derived plasma eicosanoids demonstrated inhibitor effectiveness and specificity. Furthermore, AVX002 dose-dependently reduced kidney fibroblast production of profibrotic, pro-inflammatory components leading to progressive fibrotic kidney failure. In conclusion, our results strongly suggest that inhibition of cPLA_2_α holds promise as a novel therapeutic strategy for renal fibrosis and chronic kidney disease.

## 1. Introduction

Chronic kidney disease is a global public health problem. There is an unmet clinical need for the identification of new treatment targets and the development of novel agents acting on one or more disease mechanisms. The prevalence and associated burden of CKD is rising, with an estimated global prevalence of CKD of 10–15%. There are many recognized causes of CKD, such as vascular disease, glomerulonephritis, hypertension and diabetic kidney disease (DKD), which is the leading cause of CKD and end-stage kidney disease (ESKD) [1,2]. CKD is defined by significantly decreased kidney function, as measured by reduced glomerular filtration rate (GFR), of at least three months duration. CKD and acute kidney injury (AKI) have traditionally been considered as distinct pathologies; however, AKI and CKD are recognized to represent a continuum as AKI leads to de novo or accelerated chronic and end-stage kidney diseases and CKD is a major risk factor for the development of new AKI [3].

Progressive tubulointerstitial fibrosis is characteristic of most progressive kidney diseases, regardless of initial injury [4,5]. Fibrotic diseases are characterized by excessive deposition of extracellular matrix (ECM) constituents and, as a result, increased stiffness of the affected tissues [6]. Interstitial fibroblasts are activated and differentiate to myofibroblasts which proliferate and produce matrix material. Transforming growth factor β1 (TGF-β1) is a significant driver of fibrosis and contributor to the differentiation to myofibroblasts. Myofibroblasts are regulated by many factors, including autocrine factors and paracrine signals derived from lymphocytes and macrophages. In addition to TGF-β1, critical profibrotic factors include monocyte chemotactic protein-1 (MCP-1), tumor necrosis factor α (TNFα), and intracellular activation of nuclear factor κB (NF-κB) [4]. Inflammation plays a critical role in the initiation and progression of renal fibrosis [4]. Group IVA cytosolic phospholipase A_2_ (cPLA_2_α) is a key player regulating arachidonic acid (AA) release for eicosanoid biosynthesis and is the only enzyme among the PLA2 enzyme family members that displays high selectivity for phospholipids carrying AA at their *sn*-2 position [7]. The cyclooxygenase (COX), lipoxygenase (LOX), and cytochrome P450 (CYP450) enzymatic pathways convert released AA to biologically active, pro-inflammatory eicosanoids. While there is evidence that cPLA_2_α can contribute to fibrosis [8,9,10], a causal link has not been well established.

In this study, we characterize the impact of cPLA_2_α deletion and the selective pharmacological inhibition of cPLA_2_α on fibrosis development in two different CKD animal models: unilateral ureteral obstruction (UUO) and aristolochic acid nephropathy (AAN). We demonstrate that the cPLA_2_α inhibitor AVX002 significantly inhibits fibrosis development in both animal models. We also found similar effects in a third model, the streptozotocin (STZ)-induced diabetic nephropathy model [11]. AVX002 also inhibited TGF-β1-induced gene expression of fibrotic factors in kidney myofibroblasts. Our results suggest that cPLA_2_α plays an important role in the development of renal fibrosis and that targeted inhibition of cPLA_2_α might represent a novel treatment strategy to delay or stop the pathological fibrotic processes.

## 2. Materials and Methods

### 2.1. Reagents

AVX002 (1,1,1-trifluoro-3-(((3Z,6Z,9Z,12Z,15Z)-octadeca-3,6,9,12,15-pentaen-1-yl)thio)propan-2-one) was synthesized by Synthetica AS, Oslo [12]. DMSO and Streptozotocin were obtained from Sigma Aldrich (St. Louis, MO, USA). Aristolochic acid (A5512) was purchased from Sigma-Aldrich. SC-51089, PF-04418948, L-798,106, L-161,982, Gö 6983, SB203580, Nordihydroguaiaretic acid (NDGA), PD 146176, Zileuton, and ML355 were purchased from Cayman Chemical (Ann Arbor, MI, USA). AVX002 was formulated in 100% DMSO for intraperitoneal (ip) administrations. For subcutaneous (sc) administrations, AVX002 was formulated in a sodium citrate buffer containing Polysorbate 80 and MCT oil. All AVX002 solutions were sterilized by filtration and saturated with Argon 5.0 AGA prior to aliquoting in tubes stored at −80° C until further use.

### 2.2. Antibodies

Anti-Collagen I (rabbit polyclonal, ab34710) and F4/80 (rat monoclonal, Clone T45-2342) were obtained from BD Biosciences. Anti-Fibronectin (1:25 dilution, rabbit polyclonal, ab2413 from Abcam (Cambridge, MA, USA)), Anti-TGFβ1 (1:100 dilution, rabbit polyclonal, ab92486 from Abcam), and Anti-αSMA (1:100 dilution, rabbit polyclonal, NB600-531) were obtained from Novus. P-Smad3 (rabbit monoclonal [EP823Y], ab52903 from Abcam), Smad3 (rabbit polyclonal, ab84177 from Abcam), α-SMA (rabbit polyclonal, ab5694 from Abcam), Fibronectin (rabbit polyclonal, ab2413 from Abcam), GAPDH (rabbit polyclonal, ab9485 from Abcam), and ERK (rabbit polyclonal, sc-154 from Santa Cruz (Santa Cruz, CA, USA)) were also used. Other reagents used for immunocytochemistry were EnVision FLEX Wash buffer (K8007, Agilent Dako (Santa Clara, CA, USA)), Target Retrieval Solution (HIER) low pH6 (K8005, Agilent Dako), and Antibody diluent (S2022, Agilent Dako (Santa Clara, CA, USA)).

### 2.3. Animals

Generation of *GIVAPLA_2_* KO mice was previously described [13]. Mice (male) were maintained under specific-pathogen-free conditions. The mice were given a standard rodent chow and water ad libitum. All animal care and experimental protocols were performed according to animal experimental procedures approved by the local animal care committees.

Unilateral ureteral obstruction (UUO) on control and cPLA_2_α knockout mice: UUO surgery was performed by making a retroperitoneal incision under intraperitoneal pentobarbital (50 mg/kg) anesthesia. The left ureter was dissected free and ligated with 5.0 silk in age-matched WT and *cpla_2_α* KO mice. Incision and suture without kidney ligation were performed in animals serving as sham controls. At the time of sacrifice, animals were perfused via the left ventricle with 30 mL of PBS for 2 min, and then kidneys were perfusion-fixed with PLP (2% paraformaldehyde, 75 mM L-lysine, 10 mM sodium periodate) for preservation for histological and immunohistochemical studies. Other kidneys were snap-frozen in liquid nitrogen and subsequently used for biochemical analysis.

UUO/AVX002 study: Female C57BL/6J mice were obtained from Japan SLC, Inc. (Hamamatsu, Shizuoka, Japan). The mice were 7 weeks old at the initiation of the study. On day 0, UUO surgery was performed. The abdomen was cut open, and the left ureter was exteriorized. The ureter was ligated with 4-0 nylon sutures at two points. The peritoneum and the skin were closed with sutures. The mice were treated with AVX002 or vehicle (citrate buffer containing Polysorbate 80) administered sc from day 0. AVX002 was administered sc daily, at 3 and 6 mL/kg (6 or 12 mg/kg). The animals were sacrificed on day 14 after UUO surgery and samples were collected for further analysis.

Aristolochic acid nephropathy (AAN): Male BALB/c mice (average weight: 22.5 g/each) were obtained from Charles River. On day 0, 5 mg/kg AA was administered intraperitoneally. In some animals, AVX002 (4 mg/kg or 8 mg/kg) was administered 1 h before AA and daily for 4 days and then once every other day. Blood and urine were sampled on day 2. Animals were sacrificed and blood and kidney tissue taken on day 28.

STZ/AVX002 study: Male Sprague-Dawley (Hsd) rats were obtained from Harlan Laboratories (Houston, TX, USA). Rats were approximately 6 weeks old at the initiation of treatment (body weight range of 160 and 180 g). Diabetic nephropathy was induced in rats by tail-vein injection of STZ (S0130; Sigma Aldrich Corporation, St. Louis, MO, USA) at a dose of 50 mg/kg in 0.4–0.45 mL of citrate buffer. Sham controls received a similar injection in citrate buffer in the absence of STZ. The injections were given on day 0. Treatment started on day 3 and continued for up to 6 weeks. During the study, and to confirm blood glucose elevation induced by STZ, a drop of blood from the freshly pierced tip of the tail was collected using a sterile 25G needle. Blood glucose measurements were conducted immediately by direct test strip analysis using a ReliOn Ultima Glucometer (Abbott Diabetes Care Inc., Alameda, CA 94502, USA). AVX002-IP-treated groups (3 and 6 mg/kg) were injected intraperitoneally (ip) daily for the first 5 days followed by treatments every second day. Vehicle-treated animals were administered citrate buffer-based vehicle daily for the first 5 days followed by treatments every second day. The animals were sacrificed 6 weeks after experiment initiation, approximately 5 h after the last AVX002 or vehicle dose and collection of samples for urine analysis.

### 2.4. Serum Creatinine

All blood samples were obtained from the tail vein and placed in collecting tubes. Blood from mice was then centrifuged, and serum creatinine was measured by Isotope Dilution LC-MSMS at the UAB/UCSD O’Brien Center core.

### 2.5. Urine Chemistry

STZ study, urine protein: The rats were maintained in metabolic cages for urine collection. Measurements of urine protein were performed in 12 h volumes ranging from 7 to 16 mL. All 12 h urine protein values were extrapolated to 24 h. Protein was measured using the Bradford assay kit (Quick Start© Bradford dye reagent and BSA standard set, cat. #500-0205; Bio-Rad Laboratories, Inc., Hercules, CA, USA). The kit is based on a colorimetric assay measuring the reaction of proteins with an alkaline copper tartrate solution and Folin reagent.

STZ study, urine creatinine: The Creatinine Companion Assay Kit (catalog #1012; Exocell, Inc., Philadelphia, PA, USA) was used to measure urine creatinine. The procedure is an adaptation of the alkaline picrate method and entails the determination of the differential absorbance in a sample before and after the addition of acid to correct for color generation due to interfering substances. A Bio-Rad iMark microplate reader was used for the analysis of all samples.

### 2.6. Eicosanoid Profiling in Plasma

Free eicosanoid levels in plasma from the AAN study in mice were determined by quantitative UPLC-MS/MS using the detailed method described elsewhere [14] based on the original LIPID MAPS. Specifically, plasma aliquots were spiked with a mixture of 26 internal deuterated standards and intensities were compared with standard curves run on 136 primary standards of eicosanoids and related oxylipins. Plasma aliquots containing the internal standards in 10% ethanol were subjected to solid-phase extraction (SPE) using Strata-X polymeric reversed-phase columns and methanol elution. Dried samples were taken up in buffer consisting of water/acetonitrile/acetic acid and analyzed on a Waters Accuity UPLC and a Sciex 6500 Triple Quad mass spectrometer, AB Sciex, Framingham, MA 01701 and the quantities of each eicosanoid consistently observed above the threshold baseline were determined [14].

### 2.7. Kidney Hydroxyproline Content

UUO, *GIVAPLA_2_* KO mice: To estimate total kidney collagen content, hydroxyproline was measured. Each weighed kidney sample was hydrolyzed in 6 N HCl for 18 h at 110 °C and dried by speed vacuum centrifugation for 2 h. Dried samples were solubilized in a citric acid buffer (0.23 mol/L citric acid, 0.88 mol/L sodium acetate trihydrate, 0.85 mol/L sodium hydroxide, and 1.2% acetic acid) and filtered through 0.45 µM centrifugal filter units (Ultrafree MC) (Millipore Co., Burlington, MA 01803, USA). Samples diluted in citric acid buffer (20 µL) were loaded into each well of a microtiter plate, followed by 100 µL of chloramine T solution (1.4% chloramine T and 10% n-propanol in citric acid buffer). After a 15 min incubation at room temperature, 100 µL of Erlich’s solution (15% p-dimethylaminobenzaldehyde, 62% n-propanol, and 18% perchloric acid) was added and the mixture incubated for 20 min at 65 °C. Optical density was read at 550 nm and the amount of hydroxyproline was determined against a standard curve generated using known concentrations of hydroxyproline (Sigma Chemical Co. St. Louis, MO, USA). Results were expressed as µg hydroxyproline/mg kidney dry weight.

UUO/AVX002: Frozen left kidney samples were processed by an alkaline–acid hydrolysis method. Kidney samples were dissolved in 2 N NaOH at 65 °C and autoclaved at 121 °C for 20 min. The lysed samples (400 µL) were acid-hydrolyzed with 400 µL of 6 N HCl at 121 °C for 20 min and neutralized with 400 µL of 4 N NaOH containing 10 mg/mL activated carbon. AC buffer (2.2 M acetic acid/0.48 M citric acid, 400 µL) was added to the samples, followed by centrifugation to collect the supernatant. A standard curve of hydroxyproline was constructed with serial dilutions of trans-4-hydroxy-L-proline (Sigma-Aldrich, St. Louis, MI, USA) starting at 16 µg/mL. The prepared samples and standards (each 400 µL) were mixed with 400 µL chloramine T solution (Wako Pure Chemical Industries, Osaka, Japan) and incubated for 25 min at room temperature. The samples were then mixed with Ehrlich’s solution (400 µL) and heated at 65 °C for 20 min to develop the color. After samples were cooled on ice and centrifuged to remove precipitates, the optical density of each supernatant was measured at 560 nm. The concentrations of hydroxyproline were calculated from the hydroxyproline standard curve. Protein concentrations of kidney samples were determined using a BCA protein assay kit (Thermo Fisher Scientific, Waltham, MA, USA) and used to normalize the calculated hydroxyproline values. Results were expressed as µg hydroxyproline/mg total protein.

### 2.8. Immunohistochemistry

UUO, *GIVAPLA_2_* KO mice, and AAN: After they were perfusion-fixed with PLP (2% paraformaldehyde, 75 mM L-lysine, 10 mM sodium periodate), kidneys were excised and placed in PLP overnight at 4 °C. They were then washed and stored in PBS containing 0.02% sodium azide at 4 °C. Fixed tissue was embedded in paraffin and cut into 4-micron sections using a microtome. Sections were mounted on microscope slides and stored at room temperature until use. The sections were deparaffinized with xylene and rehydrated with 100, 95, and 80% ethanol. To block the activation of endogenous peroxidase the tissue was treated with 2% H_2_O_2_ in methanol and washed with PBS. To unmask antigens, the slide was microwaved with an unmasking solution (Vector) and washed with PBS. The sections were blocked with 2% normal serum according to the host species of secondary antibodies for 30 min.

UUO/AVX002: Kidneys were fixed in 10% neutral buffered formalin (Wako Pure Chemical Industries) and embedded in paraffin. Collagen deposition in kidney sections was visualized by Picrosirius Red staining (Waldek, Germany). For quantification of interstitial fibrosis area, brightfield images in the corticomedullary region were captured using a digital camera (DFC295) at 200-fold magnification, and the positive areas in 5 fields/section were measured using ImageJ software (version 1.54r, National Institute of Health, Bethesda, MD, USA).

To visualize α-SMA, fibronectin, and TGF-β1 deposition, sectioning and immunohistochemistry were performed by the Cellular and Molecular Imaging Core Facility (CMIC), Norwegian University of Science and Technology (NTNU). Tissue was cut in 4 µm thick sections, dried, deparaffinated, and rehydrated. After heat-induced antigen retrieval (HIER, 20 min), the endogenous peroxidase activity was quenched with peroxidase block, and the primary antibody was incubated for 60 min at room temperature. Immunohistochemical reactions were visualized with Dako Rabbit Envision + HRP with DAB+ as chromogen (K4011, Agilent Dako, Santa Clara, CA, USA). Sections were counterstained with hematoxylin (CellPath, Newtown, Powys, UK). Brightfield images of the kidney sections were captured using the EVOS FL Auto 2 Imaging System equipped with a color camera and a 20×/0.45 objective by mosaic tiling. Quantitative analysis of the antibody staining was performed using CellProfiler image analysis software [15,16]. Any images with white regions (corresponding to the edge of the tissue section or rips or holes in the tissue) were excluded from the analysis. Filters were applied to unmix the DAB staining, and a threshold was applied to identify positively stained areas. The total intensity of the positively stained areas was measured for each image and the average staining intensity per kidney was determined based on a minimum of 4 (sham) or 6 (UUO-treated) images. Data are the mean +/− SEM for 10 animals (sham), 15 (vehicle), 8 (AVX002 6 mg/kg) and 14 animals (AVX002 12 mg/kg). One-way ANOVA and Dunnett’s multiple comparisons test were performed using GraphPad Prism version 7.03 for Windows (GraphPad Software, La Jolla, CA, USA).

### 2.9. Enzyme-Linked Immunoassay Detection

Cell supernatant samples were analyzed by enzyme-linked immunosorbent assay (EIA) for PGE_2_ (Cayman No514010) and 6-keto PGF_1α_ (Cayman No515211) according to the kit protocols. Cell supernatants were assayed undiluted for PGE_2_ and in 1:3 dilution for PGF_1α_. Supernatants were hybridized with overnight incubation, and enzymatic conversion of the substrate was read at OD420 nm. Data were processed using a 4-parameter logistic fit model.

### 2.10. Western Blotting

Equal amounts of protein samples were separated on 10%, 12.5%, or 15% polyacrylamide gels and transferred to a nitrocellulose membrane (Amersham Pharmacia, Piscataway, NJ, USA). Blots were incubated with primary antibodies overnight. After being washed, blots were incubated with a 1:4000 dilution of a secondary antibody for 1 h. Blots were developed with an ECL detection system (PerkinElmer, Waltham, MA, USA).

### 2.11. Cell Culture

NRK-49F (normal rat kidney fibroblasts, ATCC^®^ CRL-1570™) were maintained in DMEM with 4500 mg/L glucose (Billerica, MA, USA), 5% FBS, L-glutamine, and gentamicin. For experiments, 3 × 10^5^ cells/well were seeded in 6-well plates. After three days, post-confluent cells were serum-starved for 24 h. They were then pre-treated with inhibitors for 90 min before they were treated with 10 ng/mL TGF-β1 (Minneapolis, MN, USA) for 24 h unless otherwise stated in the figure legend.

### 2.12. qPCR

UUO, *cpla_2_α* KO mice: Total RNA was isolated from 10–15 mg renal frozen tissue using TRIZOL (Invitrogen, Carlsbad, CA, USA). RNA samples were quantified by spectrophotometry (Wilmington, DE, USA) and converted to DNA using oligo-dT. Quantitative real-time PCR was performed on an iCycler iQ (Hercules, CA, USA), using a real-time PCR assay kit (TakaRa, Dalian, China). Primer sequences are listed in Table 1.

UUO/AVX002: Total RNA was extracted from kidney samples using RNAiso (Takara Bio, Tokyo, Japan) according to the manufacturer’s instructions. A quantity of 1 µg of RNA was reverse-transcribed using a reaction mixture containing 4.4 mM MgCl_2_ (F. Hoffmann-La Roche, Basel, Switzerland), 40 U Rnase inhibitor (Toyobo, Osaka, Japan), 0.5 mM dNTP (Promega, Madison, WI, USA), 6.28 µM random hexamer (Promega), 5x first strand buffer (Promega), 10 mM dithiothreitol (Invitrogen, Carlsbad, CA, USA) and 200 U MMLV-RT (Invitrogen) in a final volume of 20 µL. The reaction was carried out for 1 h at 37 °C, followed by 5 min at 99 °C. Real-time PCR was performed using real-time PCR DICE and SYBR premix Taq (Takara Bio). Primer sequences are listed in Table 2.

NRK-49F: Total RNA was isolated using the RNeasy Mini Kit (Qiagen, Duesseldorf, Germany). cDNA synthesis was performed with 1 µg total RNA in a 20 µL reaction using the QuantiTect Reverse Transcription Kit (Qiagen). After synthesis, cDNA was diluted 1:6 with RNase-free water. qPCR was performed with LightCycler 480 SYBR Green I Master (Roche, Basel, Switzerland), and the qPCR analyses were carried out using the Lightcycler 96 system (Roche). Primer sequences are listed in Table 3.

### 2.13. Nephroseq Expression Database

Nephroseq datasets (https://nephroseq.org; University of Michigan, Ann Arbor, MI, USA) were queried for overexpression of different genes using the appropriate search functions and export result methodologies available in the database. The search function allows for the introduction of selection criteria such as minimum fold overexpression, e.g., >1.5-fold, and r correlation coefficient values of at least 0.5 and a minimum significance level of *p* < 0.05 were used for selection.

### 2.14. Statistical Analysis

UUO, *cpla_2_α* KO mice: All results were expressed as mean ± S.E. Differences among groups were evaluated by ANOVA and between groups by Student’s *t*-test. Differences were considered statistically significant at a *p* < 0.05.

UUO/AVX002: Statistical analyses were performed using the Bonferroni Multiple Comparison Test on GraphPad Prism 6 (GraphPad Software, USA). *p* values < 0.05 were considered statistically significant. A trend or tendency was assumed when a one-tailed *t*-test returned *p* values < 0.1. Results are expressed as mean ± SD.

STZ (Probetex): Data of groups were examined by one-way analysis of variance, and individual groups were then compared with Student’s unpaired *t*-test. *p* values < 0.05 were considered statistically significant. Results are expressed as mean ± SD.

AAN: Results shown are mean ± SD. Student’s *t*-test was used; *p* values < 0.05 were considered statistically significant.

NRK-49F: Results shown are mean ± SD of three biologically independent experiments. Student’s *t*-test or one-way ANOVA was used; *p* values < 0.05 were considered statistically significant.

## 3. Results

### 3.1. cPLA_2_α Knockout Mice Are Protected from Renal Fibrosis in the Unilateral Ureteral Obstruction (UUO) Model

**Figure 1:**
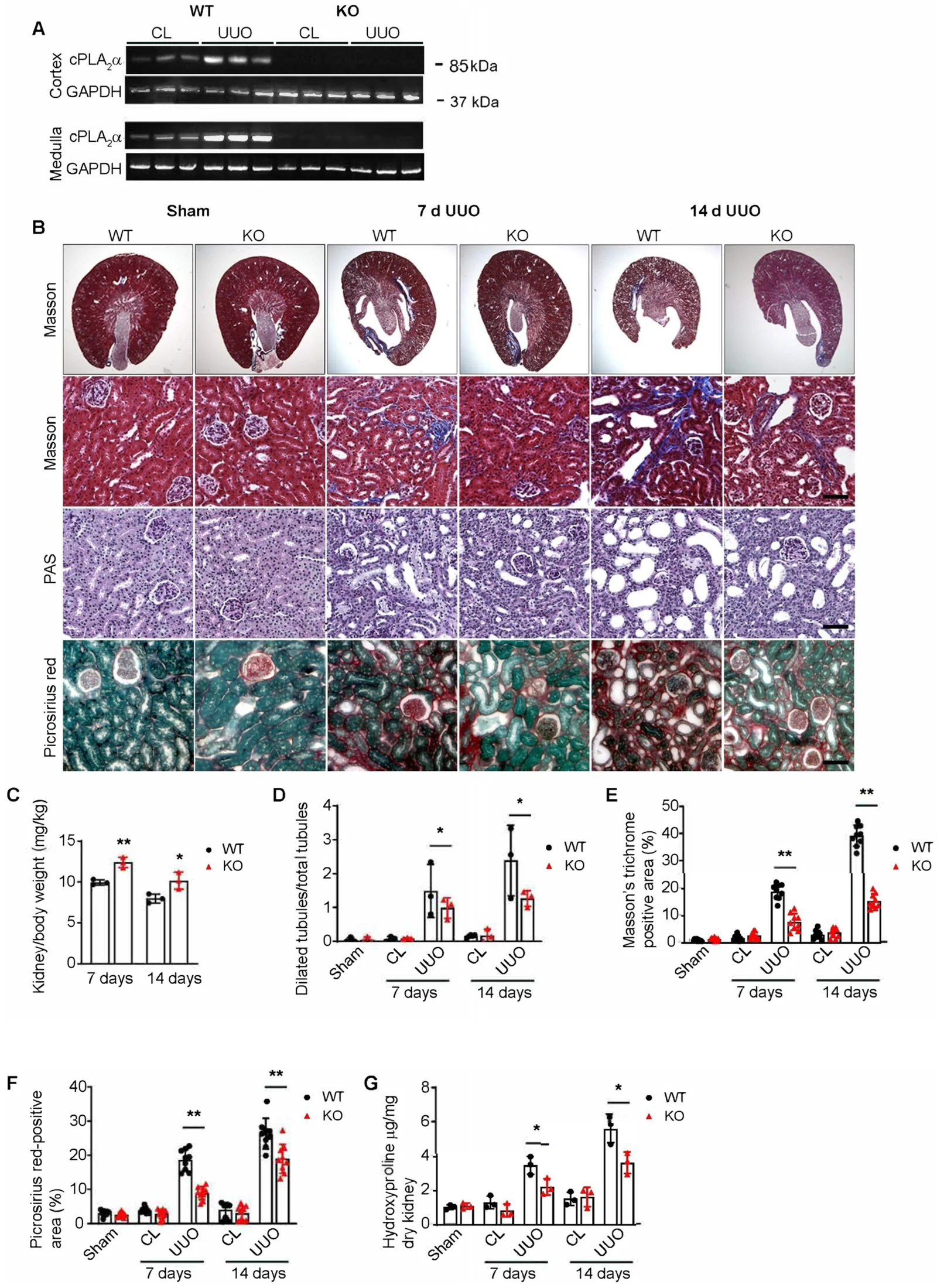

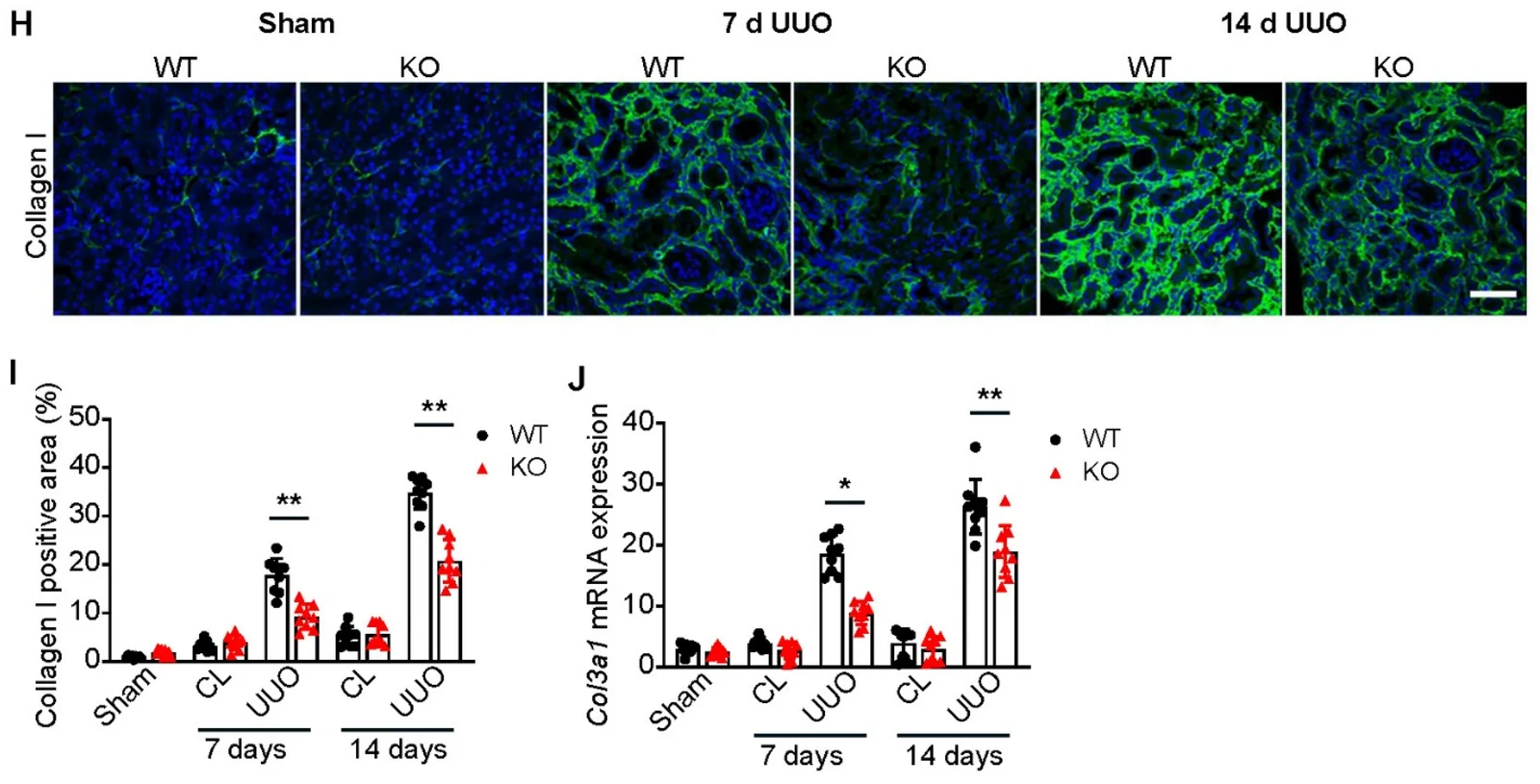
UUO-induced kidney fibrosis is attenuated in *GIVAPLA_2_* KO mice. (**A**) cPLA_2_α mRNA levels in cortex and medulla of obstructed and unobstructed kidneys of WT and *GIVAPLA_2_* KO mice were compared by RT-PCR. GAPDH was used as internal loading control. (**B**) Representative images show the cross-section of the sham and obstructed kidneys from WT and *GIVAPLA_2_* KO mice. Kidney sections were stained with Masson’s trichrome, PAS, and Picrosirius red. The blue color in Masson’s trichrome staining shows extracellular collagen deposition. Scale bar, 50 µM. (**C**) Ratio of the obstructed kidney to the whole body at 7 and 14 days UUO of WT and *GIVAPLA_2_* KO mice. (**D**) The scores for tubular dilatation of the obstructed kidney on days 7 and 14 after UUO based on PAS staining from (**B**). (**E**) Quantitative analysis of Masson’s trichrome-positive areas of sham, contralateral (CL) and UUO kidneys in WT and *GIVAPLA_2_* KO mice. (**F**) Picrosirius red-positive areas were quantified in CL and UUO kidneys. (**G**) Total kidney collagen content measured by the hydroxyproline assay on days 7 and 14 after UUO. (**H**) Immunohistochemistry for collagen I in kidneys from WT and *GIVAPLA_2_* KO mice. Scale bar, 50 µm. (**I**) Quantitative analysis of collagen I-positive areas of sham, contralateral (CL) and UUO kidneys in WT and *GIVAPLA_2_* KO mice. (**J**) *Col3a1* and *Col4a1* mRNA levels in obstructed and unobstructed kidneys of WT and *GIVAPLA_2_* KO mice were quantified by qRT-PCR and normalized to 18S mRNA content. * *p* < 0.05, ** *p* < 0.01.

As an initial step to understand the role of cPLA_2_α in the progression of renal fibrosis, we compared fibrosis development in wild-type (WT) and cPLA_2_α knockout (KO) mice in a UUO model. First, we examined the cortical and medullary expression of *GIVAPLA_2_* mRNA in contralateral (CL) and UUO kidneys by RT-PCR. UUO induced a significant increase in *GIVAPLA_2_* mRNA expression in both the cortex and medulla in WT mice, while no *GIVAPLA_2_* mRNA was detected in the kidneys of *GIVAPLA_2_^−/−^* mice (Figure 1A). This shows that *GIVAPLA_2_* is induced in response to UUO in WT mice.

Next, to explore the role of cPLA_2_α in the pathogenesis of renal fibrosis, kidney morphology and collagen deposition were examined. Kidney morphology and histologic structures were vastly altered by UUO, with a markedly reduced volume of renal parenchyma (Figure 1B, Masson panels) and a significant reduction in kidney weight (obstructed side)/body weight ratio in WT mice compared with *GIVAPLA_2_^−/−^* mice (Figure 1C). Periodic acid–Schiff (PAS) staining showed less renal structural destruction in *GIVAPLA_2_^−/−^* mice than in WT mice, as indicated by a reduction in tubular dilation and atrophy, tubular epithelial cell sloughing, and tubular basement membrane thickening (Figure 1B, PAS panel). The percentage of dilated tubules counted in PAS-stained sections was significantly higher in WT mice (Figure 1D). Collagen deposition in kidneys was examined by histology using Masson’s trichrome and Picrosirius red staining. Kidneys with ureteral obstruction for 7 and 14 days displayed interstitial expansion with collagen deposition, which was evidenced by an increase in Masson’s trichrome-positive areas within the tubulointerstitium in wild-type mice. By contrast, kidneys in *GIVAPLA_2_^−/−^* mice exhibited 60% less Masson’s trichrome-positive staining in the interstitium (Figure 1E, illustrating Masson’s trichrome-positive area in Figure 1B). There was a progressive increase in Picrosirius red staining in UUO from day 7 to day 14 in WT and *GIVAPLA_2_^−/−^* mice; however, Picrosirius red staining was significantly increased in UUO-treated WT mice when compared with *GIVAPLA_2_^−/−^* mice (WT mice had 15% more Picrosirius red staining at day 7 and 12% more on day 14; *p* < 0.01) (Figure 1F, quantitating Sirius red-positive area in Figure 1B). Total kidney collagen measured using the hydroxyproline assay was significantly higher in the WT mice at 7 and 14 days (by 28% and 35%, respectively) (Figure 1G). We also wanted to investigate the expression of specific collagens usually overexpressed in fibrosis. Collagen I protein expression was markedly decreased in *GIVAPLA_2_^−/−^* mice, compared to WT, when kidneys were exposed to UUO (Figure 1H,I). Because transcription is considered the rate-limiting step in collagen synthesis, we measured mRNA levels of *Col3a1* and *Col4a1*. Kidney mRNA levels of both collagens were significantly higher in WT when compared with KO mice at 7 and 14 days of UUO (Figure 1J). These data indicate that cPLA_2_α potentiates collagen expression and development of renal fibrosis in vivo.

### 3.2. Expression of the TGF-β1, Myofibroblast α-SMA and Fibronectin Is Significantly Reduced in cPLA_2_α Knockout Mice in the UUO Model

**Figure 2:**
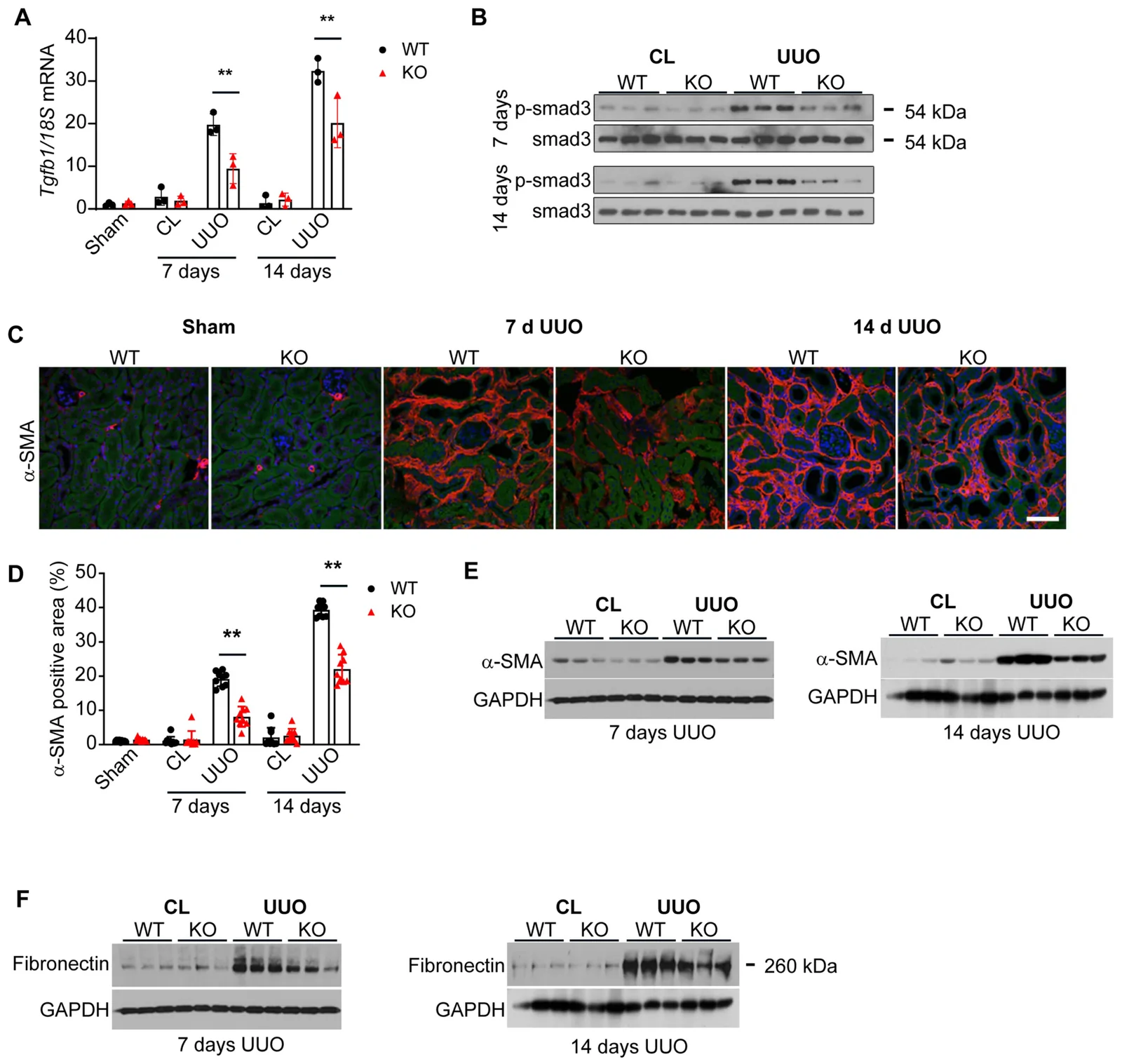
TGF-β1, α-SMA and fibronectin are enhanced in response to kidney damage, and cPLA_2_α plays an important role in these increases. (**A**) TGF-β1 mRNA levels in obstructed and unobstructed kidneys of WT and *cpla_2_α* KO mice were quantified by qRT-PCR and normalized to 18S RNA content. (**B**) Lysates of kidney tissue collected at day 7 after sham and UUO injury were subject to immunoblot analysis with specific antibodies against p-Smad3 or Smad3. (**C**) Immunohistochemistry for α-SMA in kidneys from WT and *cpla_2_α* KO mice. Scale bar, 50 µm. (**D**) Quantitative analysis of α-SMA-positive areas of sham, contralateral (CL) and UUO kidneys from WT and *cpla_2_α* KO mice. (**E**) Western blot analysis of α-SMA protein expression in 7 and 14 days obstructed and unobstructed kidneys of WT and *cpla_2_α* KO mice. GAPDH was used as internal loading control. (**F**) Western blot analysis of fibronectin protein expression at 7 and 14 days in obstructed and unobstructed kidneys of WT and *cpla_2_α* KO mice. GAPDH was used as internal loading control. ** *p* < 0.01.

To better characterize the fibrotic effects of cPLA_2_α, we next investigated the role of cPLA_2_α in regulating TGF-β1, α-smooth muscle actine (α-SMA), and fibronectin. TGF-β1 has been implicated as a profibrotic mediator in many models of fibrosis and has been proposed to play a significant role in the progression of renal fibrosis in both clinical and experimental renal disease [17]. The renal expression of *Tgfb1* mRNA in UUO kidneys was higher in WT mice compared with *GIVAPLA_2_^−/−^* mice on day 7 (50% increase) and day 14 (32% increase) (Figure 2A). No significant differences in *Tgfb1* mRNA expression were seen in sham-operated contralateral kidneys when compared with WT and *GIVAPLA_2_^−/−^* mice. Smad3 is a key downstream mediator of TGF-β1 signaling and controls the transcription of TGF-β1-responsive genes. To verify the role of cPLA_2_α in activating this signaling pathway after UUO injury, we also compared the level of phosphorylated Smad3 (p-Smad3) in WT and *GIVAPLA_2_^−/−^* mice after UUO. p-Smad3 was barely detectable in the kidneys of WT and *GIVAPLA_2_^−/−^* mice without injury. After UUO, p-Smad3 increased markedly in the renal tissue of WT mice. In contrast, p-Smad3 increased much less in the injured kidneys of *GIVAPLA_2_^−/−^* mice (53% decrease compared to WT, 7 days) (Figure 2B). This indicates that reduced levels of cPLA_2_α resulted in both reduced TGF-β1 expression and activation of the central mediator of the TGF-β1 pathway, Smad3.

The differentiation of fibroblasts into active myofibroblasts is facilitated by TGF-β1 and is characterized by de novo synthesis of *α*-SMA. Having shown that TGF-β1 levels are reduced by *GIVAPLA_2_* knockout, we next wanted to investigate if α-SMA levels were reduced as well. By immunohistochemistry, we found that α-SMA was barely detectable in the renal tissue of sham-operated mice; however, UUO injury resulted in a marked increase in α-SMA expression (Figure 2C). Both WT and *GIVAPLA_2_^−/−^* mice displayed increased numbers of α-SMA-positive cells in the obstructed kidneys, but there were significantly fewer cells in *GIVAPLA_2_^−/−^* mice (Figure 2C,D). This finding was confirmed by analyzing kidney lysates for protein levels of α-SMA at 7 and 14 days after UUO. WT mice displayed increased levels of α-SMA protein compared to *GIVAPLA_2_^−/−^* mice (Figure 2E). Fibronectin (FN) contributes to interstitial fibrosis by regulating matrix assembly, forming a bridge between cell surface receptors and collagens and proteoglycans in the ECM [18]. We analyzed UUO KO kidney lysates for protein levels of FN at 7 and 14 days after UUO. Both WT and *GIVAPLA_2_^−/−^* mice displayed increased levels of FN, but levels were lower in *GIVAPLA_2_^−/−^* animals (Figure 2F). In summary, the findings that *GIVAPLA_2_* is induced in response to UUO and that reduced cPLA_2_α levels are associated with downregulation of the TGF-β1 signaling pathway and downstream mediators of fibrosis development support cPLA_2_α as a potential biological therapeutic target in renal fibrosis.

### 3.3. Pharmacological Inhibition of cPLA_2_α Reduces Fibrosis Development in the UUO Model

Having shown that knocking out cPLA_2_α had a major effect on the development of fibrosis in the UUO model, we next investigated if pharmacological inhibition of cPLA_2_α activity would have a similar effect. For this, mice undergoing UUO were administered the highly potent and selective cPLA_2_α inhibitor AVX002 [19].

**Figure 3:**
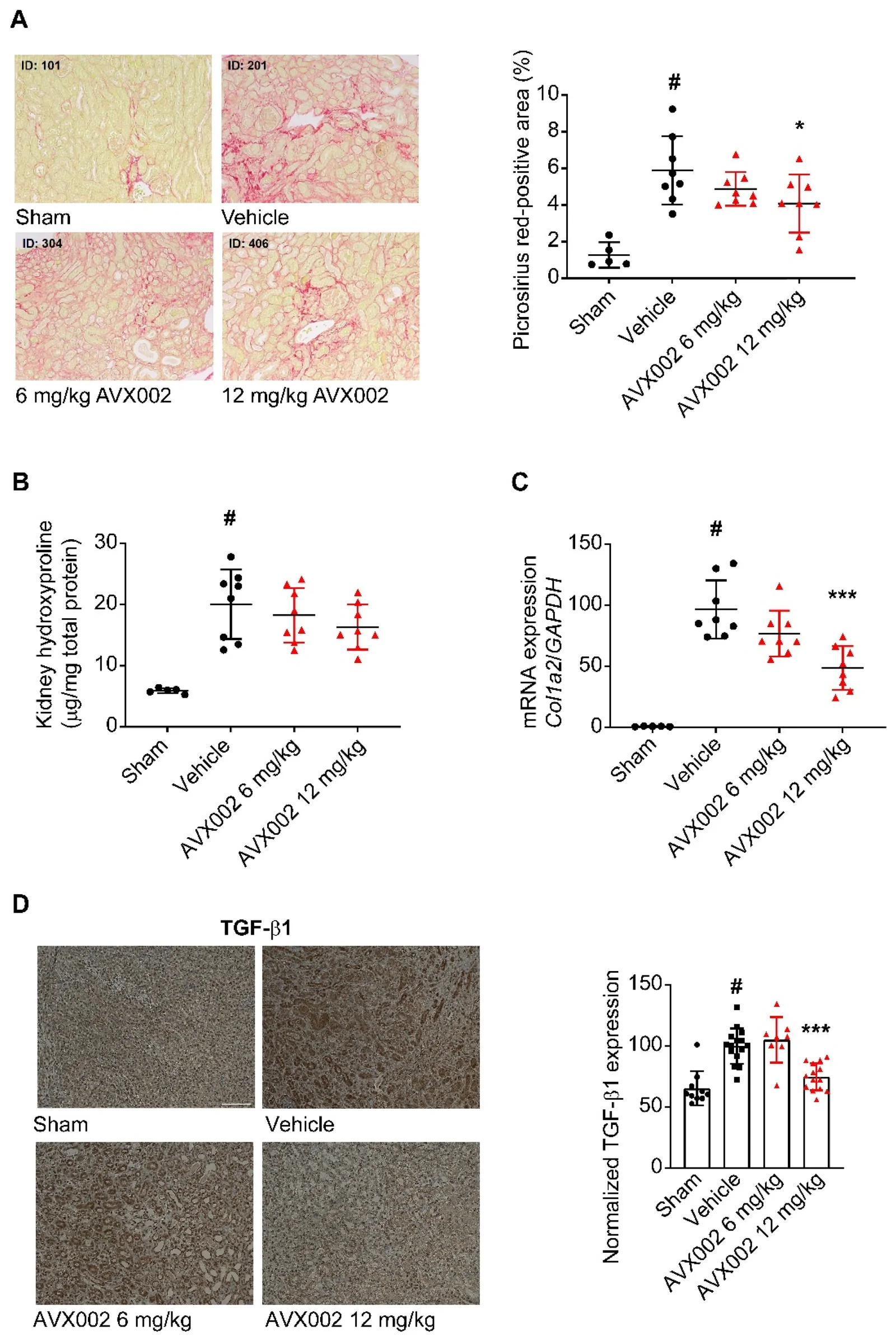

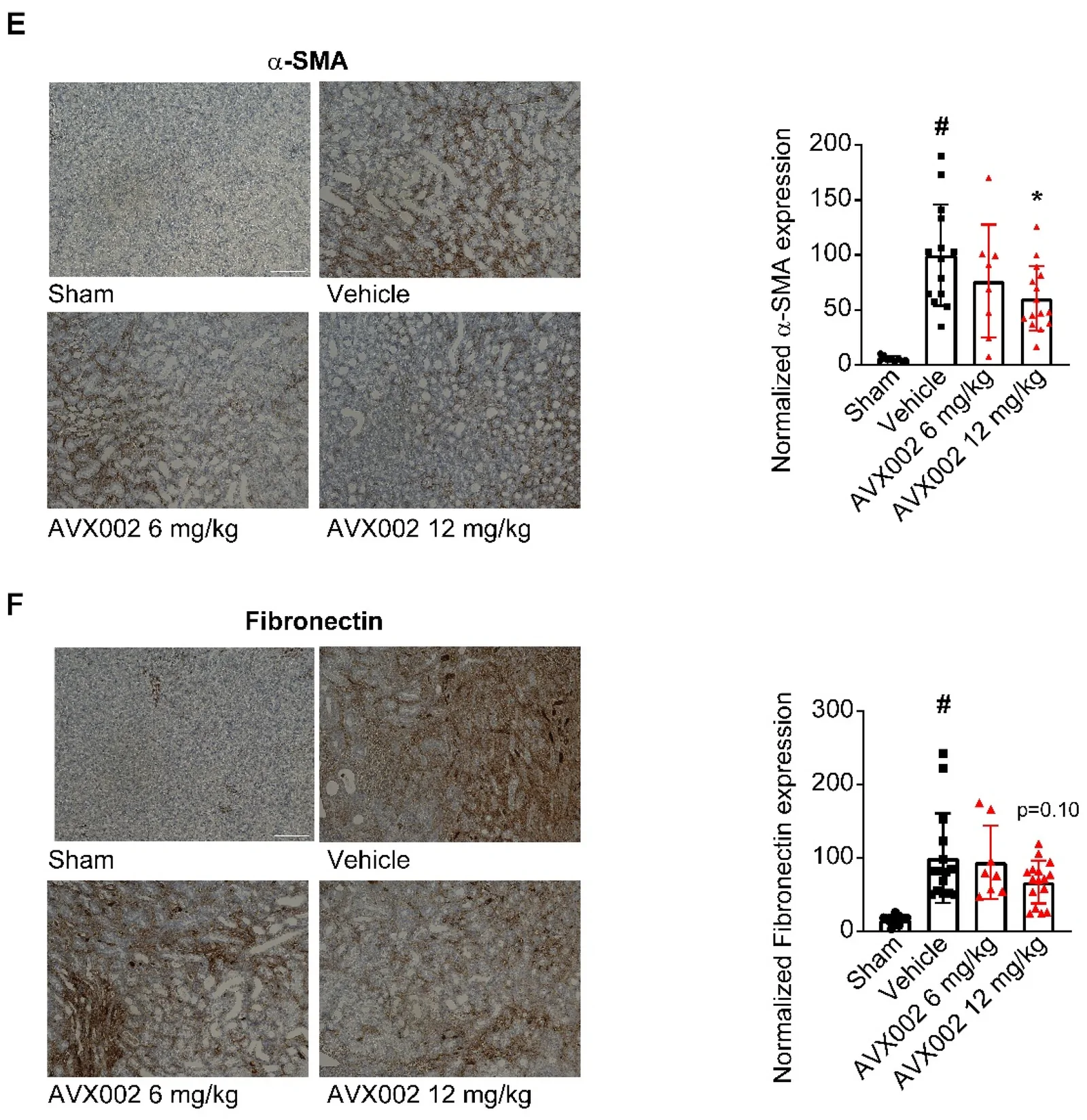
Selective inhibition of cPLA_2_α reduces fibrosis development in UUO-injured kidneys. (**A**) Representative images of picrosirius red staining in sections from UUO mice, and quantification of Picrosirius red-positive areas. (**B**) Kidney hydroxyproline content in UUO mice. (**C**) Col1a2 mRNA expression in UUO kidneys was quantified by qRT-PCR and normalized to GAPDH mRNA content. (**D**) Immunostaining for TGF-β1 in UUO kidney sections with quantification of total signal intensity (scale bar, 100 µm). (**E**) Immunostaining for α-SMA with quantification of total signal intensity (scale bar, 100 µm). (**F**) Immunostaining for fibronectin with quantification of total signal intensity (scale bar, 100 µm). Bars represent SEM. Statistical analyses were performed using one-way analysis of variance (ANOVA) with Dunnett’s post hoc test for multiple comparisons. Asterisks (*) indicate significant differences between AVX002-treated groups and the vehicle control, where * *p* < 0.05, *** *p* < 0.001. # indicates a significant difference between the vehicle treatment and sham control.

Mouse body weight decreased in all UUO mice after surgery, but there were no significant changes in mean body weight during the treatment period between the vehicle group and the treatment groups. The vehicle-treated UUO kidneys developed tubulointerstitial injury, consisting of tubular dilation and atrophy and interstitial fibrosis. Glomeruli were well preserved. Picrosirius red staining showed a significant reduction in the tubulointerstitial fibrosis area in 12 mg/kg AVX002-treated animals (inhibitors administered subcutaneously once daily) compared to vehicle-treated controls (Figure 3A). AVX002-induced dose-dependent reduction in kidney hydroxyproline was observed, although the reduction was not statistically significant (Figure 3B). Whole-kidney mRNA levels of *Col1a2* were dose-dependently reduced in response to AVX002 treatment, with a highly significant reduction in kidneys treated with AVX002 (Figure 3C). This indicates that AVX002-induced inhibition of cPLA_2_α activity mitigates the development of renal fibrosis in the UUO model.

### 3.4. Inhibiting cPLA_2_α Activity Reduces TGF-β1 Levels and Induction of Fibrotic Markers in the UUO Model

To investigate if inhibition of cPLA_2_α activity affected TGF-β1 protein levels, immunohistochemical staining of TGF-β1 in kidney sections from AVX002-treated UUO mice was performed. An increase in TGF-β1 protein levels in vehicle-treated mice compared to sham mice was observed. Treatment with 12 mg/kg AVX002 significantly reduced the levels of TGF-β1, which were indistinguishable from levels in sham-operated mice (Figure 3D). Furthermore, there was a robust increase in interstitial α-SMA expressing myofibroblasts in vehicle-treated mouse kidneys compared to sham kidneys. AVX002 significantly reduced α-SMA levels (Figure 3E). A clear increase in interstitial fibronectin staining in vehicle-treated mice compared to sham mice was also observed, and AVX002 reduced fibronectin levels, although not significantly (Figure 3F).

Taken together, the results from the UUO model, both for cPLA_2_α KO mice and mice treated with AVX002, further support the role of cPLA_2_α in fibrosis. Pharmacological inhibition of cPLA_2_α activity is sufficient to reduce fibrosis development.

### 3.5. Pharmacological Inhibition of cPLA_2_α Significantly Reduces Fibrosis in the AAN Model

Having shown similar effects of cPLA_2_α KO and pharmacological inhibition of cPLA_2_α with AVX002 in the UUO model, we also tested AVX002 in another clinically relevant CKD model. The aristolochic acid nephropathy (AAN) model can be used as a generic model of interstitial fibrosis, and it has characteristics of many types of interstitial nephropathies, for example, ischemia, Lupus nephritis, partial ureteric obstruction, analgesic nephropathy, and tubular toxicity [20].

**Figure 4:**
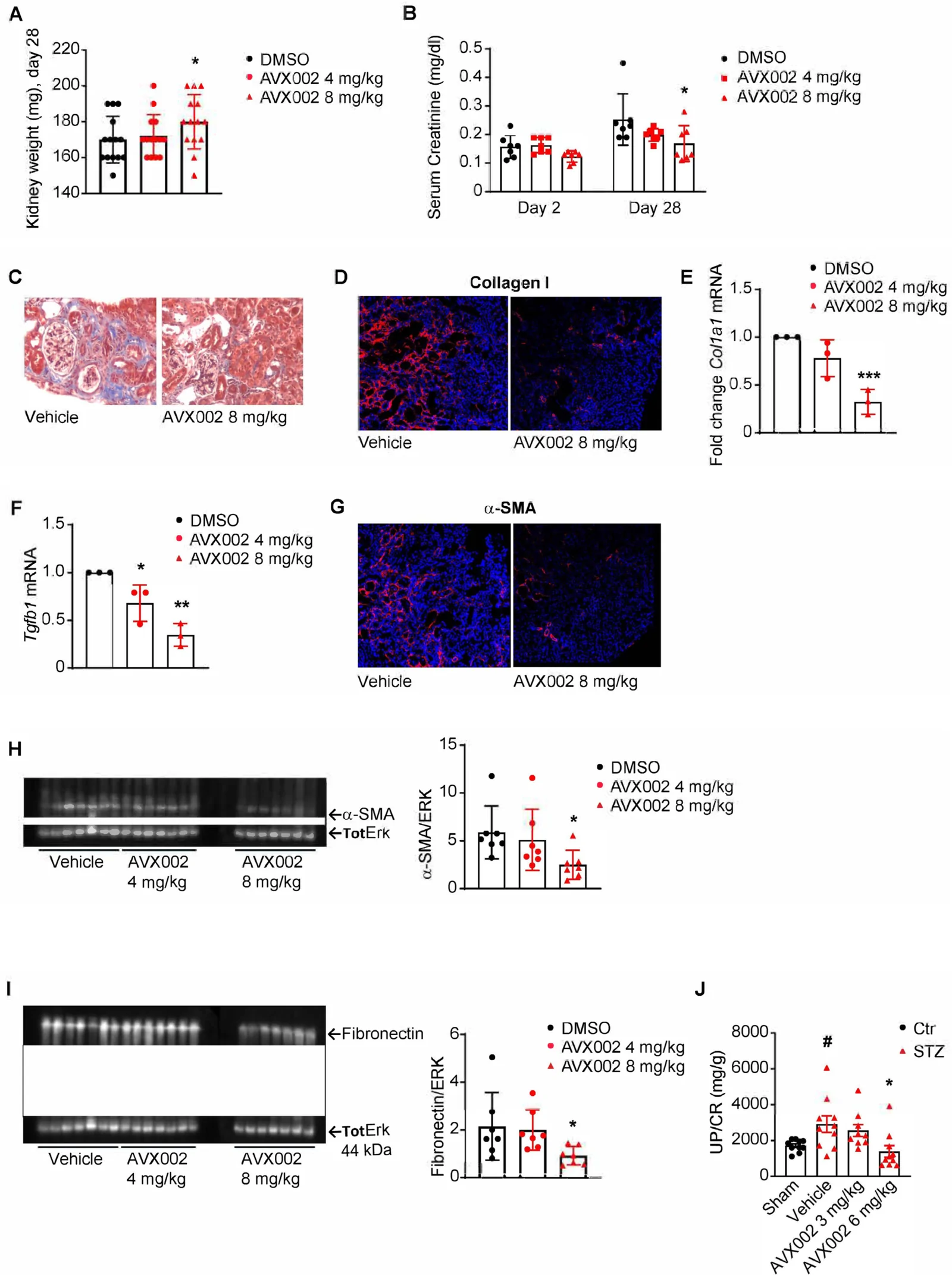
Selective inhibition of cPLA_2_α reduces fibrosis development in AAN-injured kidneys. (**A**) Kidney weight, AAN mice, day 28. (**B**) Serum creatinine levels in AAN-mice, day 2 and day 28. (**C**) Masson’s trichrome, AAN mice. (**D**) Staining collagen I in renal sections from AAN kidneys. (**E**) *Col1a2* mRNA expression in whole AAN kidneys. (**F**) *Tgfb1* mRNA from mouse AAN kidneys. (**G**) Staining of α-SMA in renal sections from AAN kidneys. (**H**) Western blot analysis of α-SMA in AAN kidney tissue. Expression levels of α-SMA were normalized to ERK1 and ERK2 (Total ERK). (**I**) Western blot analysis of fibronectin in AAN kidney tissue. Expression levels of fibronectin were normalized to Total ERK. (**J**) Urinary protein-to-creatinine ratio (UP/CR) in STZ-rats after 6 weeks. # indicates a significant difference between the vehicle treatment and sham control, * *p* < 0.05, ** *p* < 0.01, *** *p* < 0.001.

Like in the UUO model, mice treated with aristolochic acid underwent significant weight loss due to disease, but in the aristolochic acid-injected mice co-injected with AVX002 there was a strong trend of less weight loss (*p* = 0.07 for 8 mg/kg AVX002). This is consistent with the known reduction in the size of kidneys from AAN patients when compared to kidneys from healthy control subjects [21]. AAN mice treated with 8 mg/kg AVX002 had significantly larger kidneys than vehicle-treated mice, suggesting less injury (Figure 4A). As early as two days after aristolochic acid administration, there was a marked reduction in serum creatinine in AAN mice treated with 8 mg/kg AVX002 when compared to vehicle-treated mice, and this reduction was significant after 28 days relative to DMSO controls (Figure 4B). Masson’s trichrome staining showed that AVX002 treatment reduced interstitial collagen accumulation in the kidneys of AAN mice (Figure 4C). Decreased interstitial staining of collagen I was also observed in AVX002-treated kidneys compared to vehicle-treated kidneys after 28 days (Figure 4D). Quantitative evaluation by qPCR showed a dose-dependent reduction in *Col1a1* mRNA expression in response to AVX002 treatment (Figure 4E). Thus, cPLA_2_α inhibition with AVX002 reduces collagen levels and development of renal fibrosis in the AAN model, as it does in the UUO model described previously.

Aristolochic acid is also known to induce TGF-β1 expression [22,23]. There was a significant and dose-dependent reduction in *Tgfb1* mRNA in AAN kidneys with AVX002 treatment (Figure 4F). Thus, *Tgfb1* mRNA levels are reduced by AVX002 in both of our animal models, indicating that cPLA_2_α activity is important for the regulation of TGF-β1 signaling in the kidney after injury.

Like the UUO model, decreased interstitial staining of α-SMA expressing myofibroblasts was observed in renal sections from AVX002-treated AAN mice compared to vehicle-treated mice (Figure 4G). Western blot quantitative analysis of kidney tissue showed a dose-dependent effect of AVX002 on α-SMA protein expression and a significant 58% reduction in α-SMA protein in kidneys of mice treated with 8 mg/kg AVX002 (Figure 4H). Furthermore, kidney-tissue Western blots showed a dose-dependent reduction in fibronectin protein expression in response to AVX002 treatment, with a significant reduction in fibronectin in kidneys treated with 8 mg/kg AVX002 (Figure 4I). Thus, inhibition of cPLA_2_α results in significantly lower levels of α-SMA and the extracellular matrix proteins fibronectin and collagen1α2 after kidney injury.

The effect of AVX002 was also assessed in the streptozotocin (STZ) rat model, a well-known diabetic nephropathy model generated by injection of STZ, a toxin which induces pancreatic β-cell toxicity and subsequent insulin deficiency. This results in progressive elevation of blood glucose, proteinuria, albuminuria, and histopathological changes characterized by increased α-SMA and fibronectin staining relative to sham controls [24]. In this model, treatment with AVX002 resulted in a significant decrease in the urine protein-to-creatinine ratio (UP/Cr) measured after 6 weeks versus the sham control at the highest tested intraperitoneal dose of 6 mg/kg of AVX002 (*p* < 0.05, Figure 4J). Animals treated with 3 mg/kg also showed a reduction in the UP/Cr ratio, but the comparison with the vehicle treatment did not reach statistical significance (Figure 4J). A general reduction in α-SMA and fibronectin levels by AVX002 was also observed, but due to sample heterogeneity the results were not significant. Overall, and like the AAN model results, AVX002 seemed to have a protective role in this diabetic model.

### 3.6. Pro-Inflammatory Mediators, Including Eicosanoids, Are Significantly Reduced by Pharmacological AVX002 Inhibition of cPLA2α

Inflammation plays an important role in the initiation and progression of renal fibrosis [4]. To investigate if the cPLA_2_α inhibitor AVX002 affects both the fibrotic and the inflammatory components of CKD, the expression of inflammatory markers was studied.

**Figure 5:**
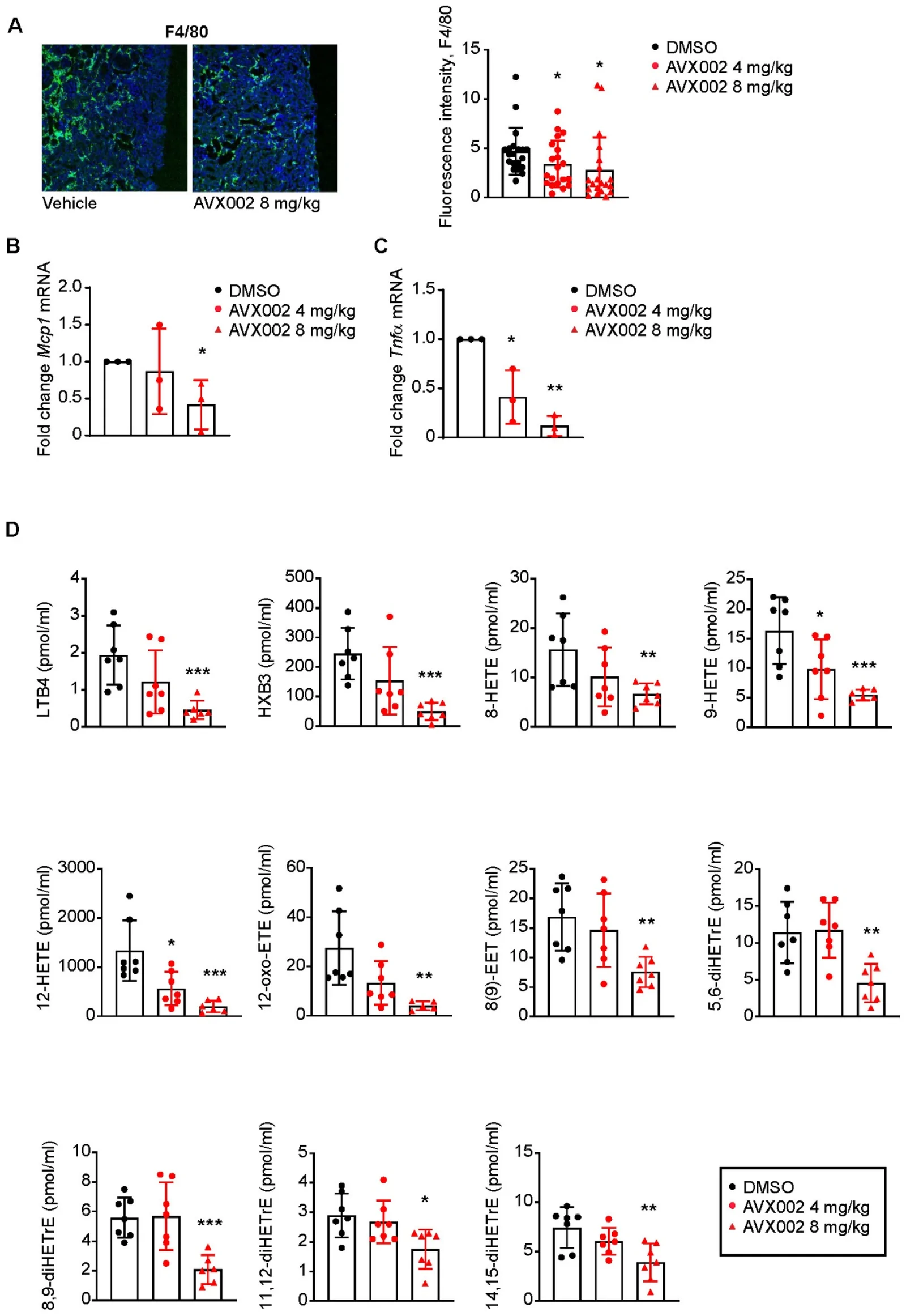
Inhibition of cPLA_2_α reduces inflammation in AAN-injured kidneys. (**A**) Staining of macrophage marker F4/80 in kidney sections from AAN mice. (**B**) MCP1 mRNA expression in whole-kidney lysate from AAN mice. (**C**) TNF-α mRNA expression in whole-kidney lysate from AAN mice. (**D**) Quantitative UPLC-MS/MS analysis of free eicosanoids in plasma from AAN mice. * *p* < 0.05, ** *p* < 0.01, *** *p* < 0.001, comparing AVX002 treatment group with DMSO control.

In kidney sections from the AAN model, significantly decreased staining of the macrophage marker F4/80 was observed in AVX002-treated mice, illustrating less infiltration of macrophages (Figure 5A). mRNA levels of the inflammatory markers MCP-1 (Figure 5B) and TNF-α (Figure 5C) were significantly reduced by AVX002 in whole-kidney lysates.

cPLA_2_α initiates the agonist-induced release of arachidonic acid, which is metabolized to a variety of pro-inflammatory eicosanoids [7]. To determine if plasma eicosanoids are regulated by AVX002, plasma from AAN mice was analyzed by quantitative UPLC-MS/MS. Eleven arachidonic acid-derived eicosanoids were significantly and dose-dependently reduced in animals treated with AVX002: LTB4, HXB3, 8-HETE, 9-HETE, 12-HETE, 12-oxo-ETE, 8(9)-EET, 5,6-diHETrE, 8,9-diHETrE, 11,12-diHETrE and 14,15-diHETrE (Figure 5D). Non-AA-derived eicosanoids did not display this property, suggesting that the arachidonyl-selective cPLA_2_α is the responsible enzyme and that it is successfully functionally inhibited by AVX002.

Thus, in the AAN model of fibrosis, AVX002 reduces infiltration of macrophages, MCP-1 and TNF-α mRNAs, and plasma pro-inflammatory eicosanoids. As for the UUO model, our results for the AAN model also support the central role of cPLA_2_α in inflammation.

### 3.7. GIVAPLA2 Is Overexpressed in Several Human Kidney Diseases and Its Expression Is Inversely Correlated with Measured Glomerular Filtration Rates (GFRs) in Patients

The results presented in previous sections have established that cPLA_2_α plays an important role in the development of kidney inflammation and fibrosis. Based on published data showing activation and/or overexpression of the enzyme in different disease models and disorders [25,26,27,28], we sought to evaluate cPLA_2_α gene expression levels by analyzing available data in Nephroseq, a widely used, publicly available, gene expression database that combines 36 datasets from renal disease studies using a total of 2105 human and mouse samples ([29] reviewed in [30]).

Table 1 presents data generated by querying Nephroseq version 5 [29] for *GIVAPLA*_2_ overexpression based on a cut-off of 1.5-fold with a significance level of at least *p* < 0.05. From the presented data, *GIVAPLA*_2_ is overexpressed in several disease sample datasets, including Lupus nephritis, IgA nephropathy, focal segmental glomerulosclerosis, and diabetic nephropathy. Interestingly, cPLA_2_α gene overexpression was inversely correlated with GFR in three datasets from diabetic and nephrotic syndrome samples (Table 4; r > −0.5, *p* < 0.05). Querying Nephroseq for gene expression of other PLA_2_ family members such as iPLA2 (*GVIAPLA*_2_) or sPLA2 (*GIIAPLA*_2_) showed different overexpression patterns, primarily in kidney transplant patient samples. Even though additional studies assessing protein expression in patient samples are needed to confirm these gene expression results, the results suggest that cPLA_2_α may play a role in human CKD, as also supported by the genetic ablation and pharmacological inhibition studies on rodents presented in previous sections [8,9].

### 3.8. Mechanistic Studies In Vitro Demonstrate That Inhibition of cPLA_2_α in Kidney Myofibroblasts Reduces TGF-β1-Induced Fibrotic Markers

**Figure 6:**
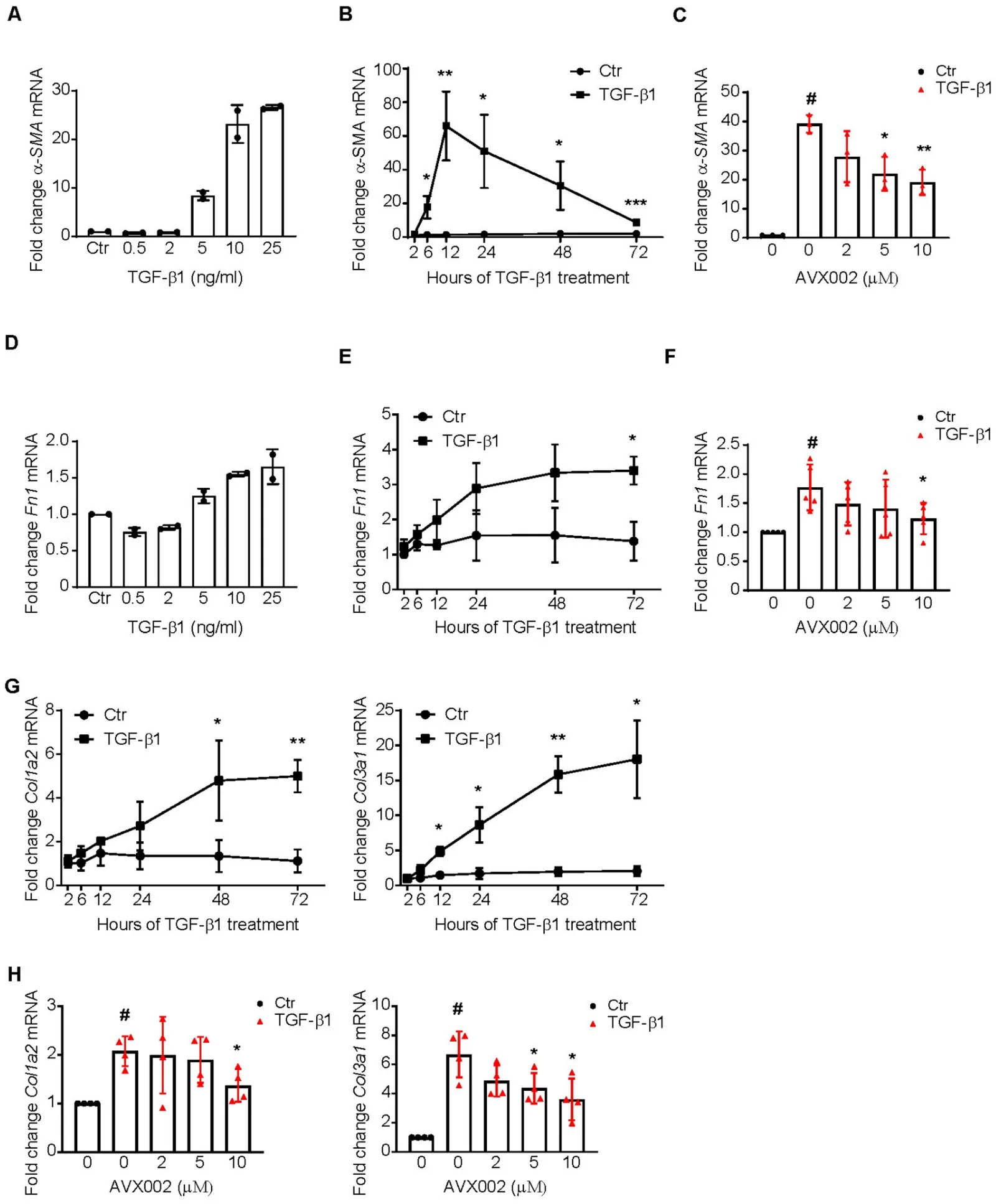
AVX002 reduces TGF-β1-induced expression of fibrotic markers. (**A**) mRNA expression of *α-SMA* in post-confluent NRK-49F cells treated with indicated doses of TGF-β1 for 24 h. (**B**) mRNA expression of *α-SMA* in post-confluent cells treated with 10 ng/mL TGF-β1 for indicated time points. (**C**) mRNA expression of *α-SMA* in post-confluent cells pre-treated with AVX002 for 90 min, before treatment with TGF-β1 (10 ng/mL, 24 h). (**D**) mRNA expression of *fibronectin* in post-confluent NRK-49F cells treated with indicated doses of TGF-β1 for 24h. (**E**) mRNA expression of *fibronectin* in post-confluent cells treated with 10 ng/mL TGF-β1 for indicated time points. (**F**) mRNA expression of *fibronectin* in post-confluent cells pre-treated with AVX002 for 90 min, before treatment with TGF-β1 (10 ng/mL, 24 h). (**G**) mRNA expression of *Col1a2* and *Col3a1* in post-confluent cells treated with 10 ng/mL TGF-β1 for indicated time points. (**H**) mRNA expression of *Col1a2* and *Col3a1* in post-confluent cells pre-treated with AVX002 for 90 min, before treatment with TGF-β1 (10 ng/mL, 24 h). Results shown are mean ± SD of three biologically independent experiments. ^#^ *p* < 0.05, * *p* < 0.05, ** *p* < 0.01, *** *p* < 0.001 for TGF-β1 vs. Ctr.

To further understand how cPLA_2_α regulates fibrosis, the rat fibroblast cell line NRK-49F was used as a model. NRK-49F fibroblasts differentiate into myofibroblasts in response to TGF-β1 [35]. TGF-β1 dose-dependently induced mRNA expression of the myofibroblast marker *α-SMA* (Figure 6A). Time-series experiments showed that *α-SMA* mRNA levels increased rapidly, with the highest expression after 12h, then gradually declined until 72h, when the expression was about four times as high as in the untreated control (Figure 6B). We examined the effect of cPLA_2_α inhibition on *α-SMA* mRNA in TGF-β1-treated myofibroblasts. Pre-treatment with AVX002 dose-dependently inhibited TGF-β1-induced expression of *α*-SMA (Figure 6C). The expression was significantly decreased by 44% with 5 µM AVX002 and by 52% with 10 µM AVX002.

The impact of TGF-β1 and cPLA_2_α on fibronectin expression in myofibroblasts was also studied. TGF-β1 dose-dependently induced *Fn1* mRNA (Figure 6D), and time-series experiments showed that *Fn1* expression steadily increased in response to 10 ng/mL TGF-β1, at least until 72 h of treatment (Figure 6E). Pre-treatment with AVX002 dose-dependently inhibited TGF-β1-induced expression of *Fn1* (Figure 6F). The expression was significantly decreased by 30% in cells treated with 10 µM AVX002. This demonstrates that inhibiting cPLA_2_α results in significantly lower levels of the mRNA for the ECM-protein fibronectin.

Time-series experiments in fibroblasts also showed that *Col1a2* and *Col3a1* mRNA expression is steadily increased in response to 10 ng/mL TGF-β1, at least until 72h of treatment (Figure 6G). *Col1a2* expression was significantly decreased by 34% with 10 µM AVX002, and *Col3a1* was significantly and dose-dependently decreased by 35% with 5 µM and 46% with 10 µM AVX002 (Figure 6H).

Taken together, the results from the cellular model support the results from the in vivo models and indicate that cPLA_2_α plays a critical role in the increase in α-SMA and the gene expression of the interstitial matrix proteins fibronectin and collagen after kidney injury both in vivo and in vitro.

### 3.9. cPLA_2_α Regulates TGF-β1-Induced Expression of Ptgs2 and Secretion of Prostaglandins in Kidney Myofibroblasts

**Figure 7:**
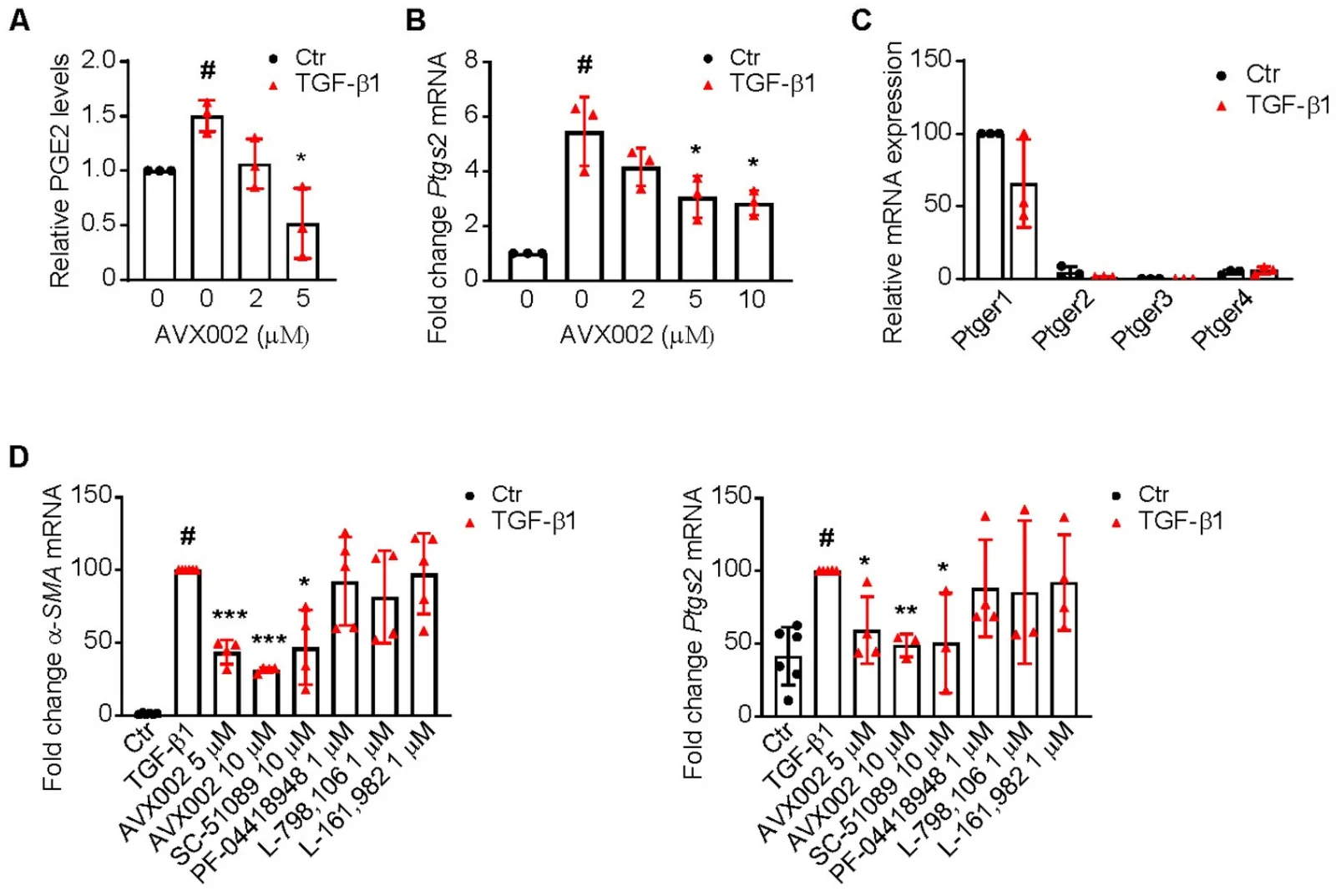
PGE2 appears to work through EP1 receptor in NRK-49F cells. (**A**) PGE2 levels in supernatant from post-confluent NRK-49F cells pre-treated with inhibitors for 90 min, before treatment with TGF-β1 (10 ng/mL, 72 h). (**B**) mRNA expression of *Ptgs2* in post-confluent cells pre-treated with AVX002 for 90 min, before treatment with TGF-β1 (10 ng/mL, 24 h). (**C**) mRNA expression of EP receptors in post-confluent cells treated with TGF-β1 (10 ng/mL, 24 h). (**D**) mRNA expression of *α-SMA* and *Ptgs2* in post-confluent cells pre-treated with inhibitors for 90 min, before treatment with TGF-β1 (10 ng/mL, 24 h). Results shown are mean ± SD of three biologically independent experiments. * *p* < 0.05, ** *p* < 0.01, *** *p* < 0.001, ^#^ *p* < 0.05 for TGF-β1 vs. Ctr.

We showed earlier that AVX002 inhibits cytokine-induced production of PGE_2_ [36,37]. To explore which cellular pathways are influenced by cPLA_2_α in myofibroblasts, NRK-49F cells were pre-treated with AVX002 and then treated with TGF-β1 for 72 h. TGF-β1-induced PGE_2_ production was dose-dependently reduced in the presence of AVX002 (Figure 7A). We also examined the effect of cPLA_2_α inhibition on *Ptgs2* mRNA encoding the COX2 protein, which is the inducible cyclooxygenase enzyme that converts arachidonic acid to prostaglandins. Pre-treatment with AVX002 dose-dependently inhibited TGF-β1-induced expression of *Ptgs2*. *Ptgs2* mRNA was significantly decreased by 44% with 5 µM and 48% with 10 µM AVX002 (Figure 7B). Thus, cPLA_2_α inhibition results in lower expression of COX2 and lower production of the prostaglandin PGE_2_, consistent with an anti-inflammatory effect of AVX002 in myofibroblasts.

### 3.10. PGE_2_ Acts via the Pro-Fibrotic Prostaglandin E_2_ Receptor EP1 in Kidney Myofibroblasts

There are four Prostaglandin E_2_ (EP) receptor subtypes, EP_1_-EP_4_, encoded by the genes *Ptger1*-*Ptger4* [38]. PGE_2_ is reported to have an anti-fibrotic effect via the EP_2_ and EP_4_ receptors and a pro-fibrotic effect via the EP_1_ and EP_3_ receptors [6]. Since AVX002, by inhibiting cPLA_2_α, leads to reduced levels of PGE_2_, and since we showed that AVX002 has a significant anti-fibrotic effect, we wanted to determine which EP receptors are expressed in the myofibroblast cells that could be involved in PGE_2_-induced signal transduction. By qPCR, EP_1_ was found to be the most abundantly expressed EP receptor isoform. We found relatively low expression levels of EP_2_ and EP_4_ receptor mRNA, and EP_3_ receptor expression was barely detected (Figure 7C). To investigate which EP receptors are most important for fibrotic signaling in myofibroblasts, we used EP receptor antagonists. Cells were pre-treated for 90 min with SC-51089, PF-04418948, L-798,160, or L-161,982, which antagonize EP_1_, EP_2_, EP_3_ and EP_4_ receptors, respectively, prior to TGF-β1 treatment. TGF-β1-induced *α-SMA* and *Ptgs2* gene expression were significantly reduced in the presence of an EP_1_ receptor antagonist, while the other antagonists did not show any significant effects, implying that of the EP receptors, the EP_1_ receptor signaling pathway is most important for *a-SMA* and *Ptgs2* gene expression in myofibroblasts (Figure 7D). Thus, our results suggest that TGF-β1-induced PGE_2_ mainly acts via the EP1 receptor to regulate *α-SMA* and *Ptgs2* in myofibroblasts.

## 4. Discussion

Chronic kidney disease is a devastating condition that is reaching epidemic levels owing in part to the increasing prevalence of obesity, diabetes mellitus, and hypertension. CKD is associated with marked increases in cardiovascular risk so that most patients will experience cardiovascular events prior to reaching ESKD [39]. While blood pressure control, disease-specific interventions, and angiotensin converting enzyme and receptor therapies have been recently supplemented with SGLT-2 and non-steroidal mineralocorticoid inhibitors and GLP-1 agonists, there continues to be risk for progression of CKD. Hence, novel therapeutic approaches to either stop or reverse progression are needed. Inflammation plays a critical role in the initiation and progression of renal fibrosis [40]. One of the most important hormones linking inflammation and fibrosis is TGF-β1, and its excessive activity leads to fibrosis, but blocking TGF-β1 may be difficult due to its pleiotropic actions [41]. Therefore, identification of novel therapeutic targets, like the cPLA2α enzyme, is welcome to hopefully regulate profibrotic, pro-inflammatory components of the pro-inflammatory process that leads to progressive fibrotic kidney failure.

In this study, our findings suggest a role for cPLA_2_α as a novel regulator of renal inflammation and profibrotic mechanisms. AVX002, a potent and selective inhibitor of cPLA_2_α, has renoprotective effects in several different animal models of kidney fibrosis. Moreover, we show that cPLA_2_α KO mice have reduced renal fibrosis. Pharmacologic cPLA_2_α inhibition also potently and significantly reduces the AA-derived plasma eicosanoids in kidney fibrosis animal models, confirming AVX002 inhibitor selectivity and role in fibrosis development. Previous studies have shown that cPLA_2_α contributes to renal dysfunction and inflammation in AngII-induced hypertension [8] and that cPLA_2_α deficiency attenuates hepatic fibrosis formation [10]. Analysis of *GIVAPLA*_2_ mRNA expression in Nephroseq [29] shows that *GIVAPLA*_2_ expression is higher in patients suffering from diabetic nephropathy and lupus nephritis, as well as patients with minimal change disease, IgA nephropathy, vasculitis, and other kidney diseases. In diabetic nephropathy, *GIVAPLA*_2_ mRNA expression is inversely correlated with estimated GFR. Thus, AVX002 inhibition of cPLA_2_α supports cPLA2α inhibition as a unique and potent therapeutic strategy.

Renal fibrosis is initiated by an injury, leading to the triggering of an inflammatory process by resident tubular epithelial cells and mesangial cells. These cells secrete pro-inflammatory cytokines and chemokines, including TNF-α, IL1-β, and MCP-1. cPLA_2_α is involved in these cellular processes and hence in the degree of infiltration of inflammatory cells, as well as in the further activation of these infiltrating cells. The infiltrated inflammatory cells produce reactive oxygen species and pro-inflammatory and fibrogenic cytokines like TGF-β, PDGF, and CTGF. This leads to the activation of pericytes with their differentiation to interstitial myofibroblasts. Myofibroblasts express α-SMA and secrete ECM proteoglycans, like fibrillar collagen type I, III, IV, and fibronectin. The increased ECM component deposits lead to the disorganization vascular rarefaction and irreversible loss of kidney function (reviewed in [42]). We show that *GIVAPLA_2_* expression is highly induced in response to acute kidney injury in the UUO model. Deletion, as well as pharmacological inhibition, of cPLA_2_α has an inhibitory effect on all these important steps in fibrosis development. cPLA_2_α inhibition results in reduced levels of *Tnfα* and *Mcp-1* mRNAs and a reduced number of macrophages in the AAN model. Inhibition or deletion of cPLA_2_α also leads to a lower number of α-SMA-expressing myofibroblasts in all models with correspondingly lower levels of the ECM proteins fibronectin and collagens. We found a AVX002 dose-dependent reduction in TGF-β1-induced α-SMA and the ECM proteins fibronectin, collagen 1a2, and collagen 3 in rat kidney fibroblasts. We conclude that cPLA_2_α plays important roles in many cell types at several levels of fibrosis development.

Small lipids such as eicosanoids regulate normal kidney function and play important roles in the pathogenesis of kidney diseases [43]. Plasma eicosanoid profiling shows that AVX002 significantly reduced the production of several arachidonic acid-derived eicosanoids in AAN mice. Both LOX and CYP450 metabolites were reduced. Also, the non-enzymatic oxidation products of arachidonic acid, 8-HETE, and 9-HETE were detected and significantly reduced in AVX002-treated animals.

Montford et al. [9] profiled eicosanoids in kidney homogenates from control and UUO tissues from WT-BMT and KO-BMT (bone marrow-silenced cPLA_2_α) animals. They showed reduced induction of PGE2, PGD2, 12-HETE, and 15-HETE in KO-BMT mice compared to WT-BMT mice. They did not detect leukotrienes in the renal tissue. However, in their subsequent paper [44] they showed that LTB_4_ is important for fibrosis development in the folic acid nephropathy model and suggested that manipulation of the 5-LO pathway may represent a treatment approach for renal fibrosis and CKD. In line with this, we show a highly significant dose-dependent reduction in serum LTB4 in response to AVX002 treatment, clearly illustrating that inhibiting cPLA_2_α reduces leukotrienes. Also, the pro-inflammatory AA-derived eicosanoid 12-HETE and its metabolite HXB3 are dose-dependently reduced in AVX002-treated mice, confirming the effect of AVX002 on cPLA_2_α and its promising potential as a treatment for fibrotic disorders and CKD.

The mouse and rat 15-lipoxygenase-1 acts primarily to convert arachidonic acid to 12-HETE (15-HETE and 12-HETE in a ratio of 1:3) [45]. 12(S)-HETE is a potent pro-inflammatory chemoattractant for neutrophils and leukocytes and induces expression of IL-6, TNFα, MCP1, and adhesion molecules in macrophages. 15-LOX mRNA and protein levels increase along with established markers of diabetic nephropathy. TGF-β1 and angiotensin II induce the expression of 15-LOX in mesangial cells, and pharmacological inhibition of 15-LOX blocks TGF-β1 and angiotensin II-induced mesangial cell hypertrophy and matrix accumulation [45]. Our result from myofibroblasts, showing that 15-LOX is important for *α-SMA* expression, seen together with the regulation of plasma 12-HETE in the AAN study, suggests that 12-HETE production, via 15-lipoxygenase-1, may be an important cPLA_2_α-mediated signaling pathway in renal fibrosis.

## 5. Conclusions

**Figure 8:**
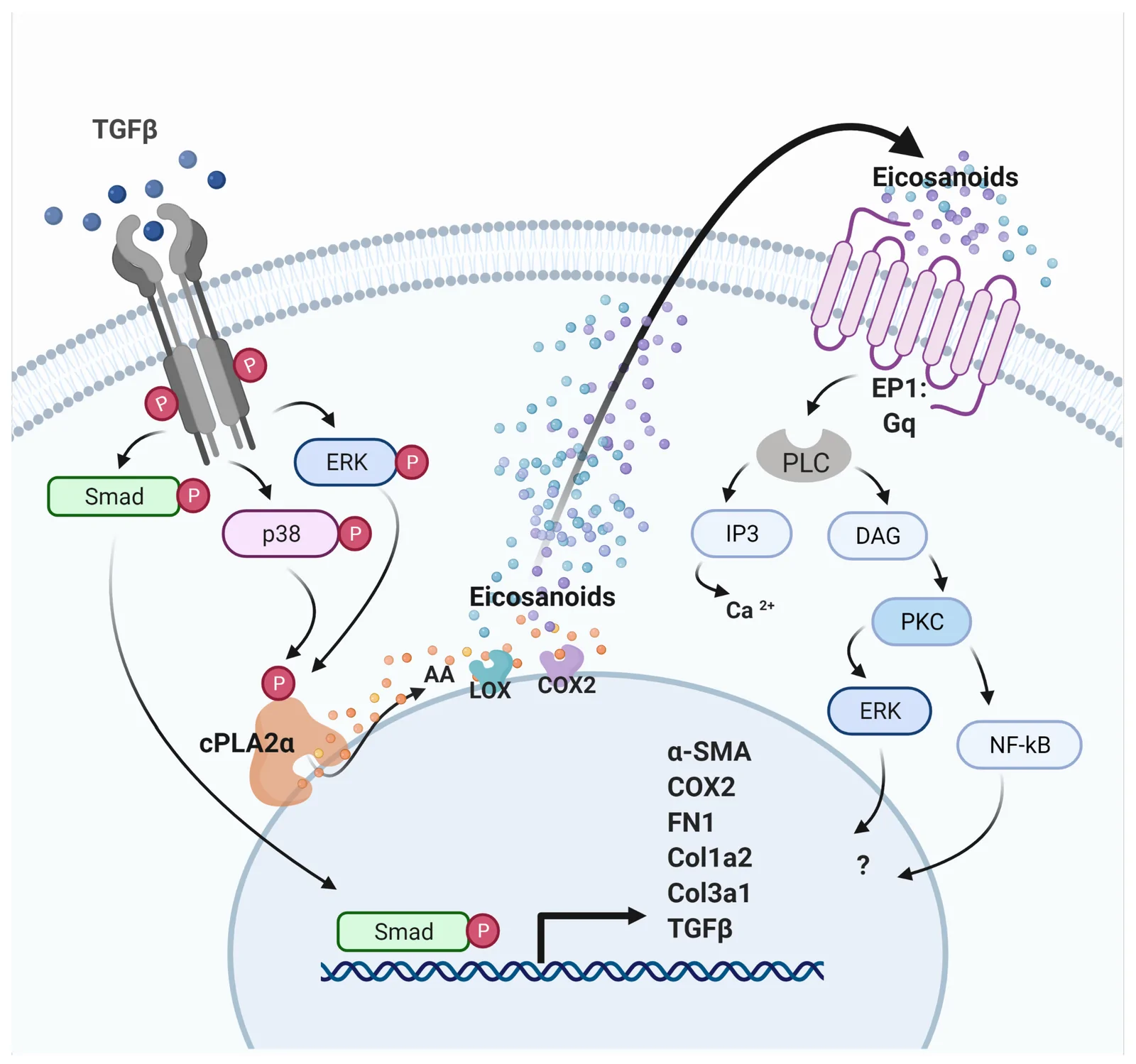
Schematic view of TGFβ-induced and cPLA_2_α-dependent autocrine pro-fibrotic signaling. TGFβ is a powerful fibrosis regulator. In the kidney fibroblast model system, TGFβ induces cPLA_2_α activation to release AA from membranes by binding to its receptor. AA released by cPLA_2_α is metabolized by LOX or COX2 to eicosanoids, which are secreted and interact with G-protein-coupled eicosanoid receptors, including PGE2 to its EP1 receptor. This leads to initiation of an intracellular kinase cascade leading to transcriptional activation of a series of pro-fibrotic markers. cPLA_2_α regulates TGFβ-induced expression of collagens, fibronectin and other factors, and therefore functions to coordinate fibrotic and inflammatory processes. Secreted pro-inflammatory and pro-fibrotic molecules can also affect surrounding cells, including other pericytes and proximal tubule cells. Created in BioRender.com.

In summary, we show that AVX002 reduces arachidonic acid-derived eicosanoids from all three enzymatic pathways: the COX, LOX and CYP450, pathways. AVX002 reduces the levels of several eicosanoids back to more basal levels, reducing their pro-inflammatory, pro-fibrotic detrimental effects (Figure 8). cPLA_2_α is a promising therapeutic target to prevent or decrease the progression of renal fibrosis.

## Figures and Tables

**Table 1:** Primer sequences: *cPLA_2_α* KO UUO and AAN study.

Oligo Name	Forward (5′-3′)	Reverse (5′-3′)
Mouse cPLA2	CTGGCCAACATCAACTTCAG	GCCAGTCTCTTCTGCATAGT
Mouse Tgf	CCACCTGCAAGACCATCGAC	CTGGCGAGCCTTAGTTTGGAC
Mouse Col1	GCTCCTCTTAGGGGCCACT	ATTGGGGACCCTTAGGCCAT
Mouse Col3	CTGTAACATGGAAACTGGGGAAA	CCATAGCTGAACTGAAAACCACC
Mouse Col4	ATCGGATACTCCTTCCTCATGC	CCAGGGGAGACTAGGGACTG
Mouse Mcp1	TAAAAACCTGGATCGGAACCAAA	GCATTAGCTTCAGATTTACGGGT
Mouse Tnf	CAGGCGGTGCCTATGTCTC	CGATCACCCCGAAGTTCAGTAG
Mouse GAPDH	CCATGGAGAAGGCTGGG	CAAAAGTTGTCATGGATGACC
Mouse 18S	GCAATTATTCCCCATGAACG	GGCCTCACTAAACCATCCAA

**Table 2:** Primer sequences: UUO/AVX002 study.

Oligo Name	Forward (5′-3′)	Reverse (5′-3′)
Mouse Col1a2	CCAACAAGCATGTCTGGTTAGGAG	GCAATGCTGTTCTTGCAGTGGTA
Mouse GAPDH	TGTGTCCGTCGTGGATCTGA	TTGCTGTTGAAGTCGCAGGAG

**Table 3:** Primer sequences: NRK-49F study.

Oligo Name	Forward (5′-3′)	Reverse (5′-3′)
Rat Acta2	GCCATCAGGAACCTCGAGAA	GGAGCATCATCACCAGCAAAG
Rat Ptgs2	CTCATACTGATAGGAGAGACG	TCGAACTTGAGTTTGAAGTG
Rat Col1a2	AGTGGAAGAGCGATTACTAC	ATTGATGGTCTCTCCTAACC
Rat Col3a1	AGTGGCCATAATGGGGAACG	CAGGGTTTCCATCCCTTCCG
Rat Fn1	GGACACTATGCGGGTCACTTGG	TGCTGTTCGTACACGCTGGAGA
Rat Ptger1	CTGGTATTGGTGGTGTTG	TAGGAGGCGAAGAAGTTG
Rat Ptger2	CCATTATGACCATCACCTTC	AAGGATGACAAAAACCCAAG
Rat Ptger3	GAAAGTTCTGCCAGATGATG	GAAATTCTCATACTCAGGGAAG
Rat Ptger4	ATCCAGATGGTCATCTTACTC	CACTTGGCTGATATAACTGG
Rat 18S	CGATTCCGTGGGTGGTGGTG	CATGCCAGAGTCTCGTTCGTTATC

**Table 4:** Nephroseq analyses. *GIVAPLA*_2_ is overexpressed in renal patient samples, and its expression is inversely correlated with GFR values in patients. The Nephroseq database version 5 [29] was queried to assess the expression of *GIVAPLA*_2_ gene in human kidney samples. Analysis of *GIVAPLA*_2_ overexpression in patient samples versus samples from healthy donors. Based on pre-selected criteria of a minimum 1.5-fold overexpression, *GIVAPLA*_2_ was overexpressed in several disease datasets in a statistically significant manner (*p* < 0.05). Diabetic nephropathy showed the highest difference, reaching approximately 3-fold in one dataset. GFR analysis. High expression of *GIVAPLA*_2_ was inversely correlated with GFR in patients (r > −0.5; *p* < 0.05). Note that datasets may include samples from several kidney diseases. ERCB: European Renal cDNA bank. GFR: glomerular filtration rate; n.p: not published yet.

Dataset	Samples	Analysis	*p* Value	Fold Change	r Value	Ref.
Berthier Lupus Glom	46	Lupus Nephritis vs. Healthy Living Donor	8.42 × 10^−11^	2.087		[31]
Ju CKD Glom	199	Lupus Nephritis vs. Healthy Living Donor	3.28 × 10^−6^	1.992		[32]
Ju CKD Glom	199	Vasculitis vs. Healthy Living Donor	1.55 × 10^−5^	1.961		[32]
Ju CKD Glom	199	Diabetic Nephropathy vs. Healthy Living Donor	1.73 × 10^−5^	2.97		[32]
Ju CKD Glom	199	Focal Segmental Glomerulosclerosis vs. Healthy Living Donor	1.37 × 10^−4^	1.709		[32]
Ju CKD Glom	199	IgA Nephropathy vs. Healthy Living Donor	7.10 × 10^−4^	1.559		[32]
Ju CKD Glom	201	Diabetic Nephropathy vs. Other Diseases	0.003	1.866		[32]
ERCB Lupus Glom	32	Lupus Nephritis vs. Healthy Living Donor	4.52 × 10^−4^	2.846		n.p.
ERCB Nephrotic Syndrome TubInt	37	Diabetic Nephropathy vs. Healthy Living Donor	0.002	2.286		n.p.
ERCB Lupus Glom	32	Minimal Change Disease vs. Healthy Living Donor	0.003	2.53		n.p.
ERCB Lupus TubInt	41	Lupus Nephritis vs. Minimal Change Disease and Healthy Living Donor	0.011	1.586		n.p.
Reich IgAN Glom	33	IgA Nephropathy vs. Healthy Living Donor	8.03 × 10^−4^	1.545		[33]
Woroniecka Diabetes TubInt	22	Diabetic Nephropathy vs. Healthy Living Donor	0.001	2.194		[34]
Woroniecka Diabetes Glom	22	Diabetic Nephropathy vs. Healthy Living Donor	0.011	1.86		[34]
Woroniecka Diabetes Glom	22	All Measured Samples	1.96 × 10^−4^		−0.713	[34]
Woroniecka Diabetes TubInt	22	All Measured Samples	6.74 × 10^−4^		−0.668	[34]
ERCB Nephrotic Syndrome TubInt	16	Focal Segmental Glomerulosclerosis Samples	0.027		−0.551	n.p.

## Data Availability

The original contributions presented in this study are included in the article/Supplementary Material. Further inquiries can be directed to the corresponding author(s).

## Supplementary Materials

The following supporting information can be downloaded at https://www.mdpi.com/article/10.3390/biom16101430/s1: Figure S1: WB original images.

## References

[1] Jefferson J.A., Shankland S.J., Pichler R.H. (2008). Proteinuria in diabetic kidney disease: A mechanistic viewpoint. Kidney Int..

[2] Levin A., Tonelli M., Bonventre J., Coresh J., Donner J.-A., Fogo A.B., Fox C.S., Gansevoort R.T., Heerspink H.J.L., Jardine M. (2017). Global kidney health 2017 and beyond: A roadmap for closing gaps in care, research, and policy. Lancet.

[3] Ferenbach D.A., Bonventre J.V. (2016). Acute kidney injury and chronic kidney disease: From the laboratory to the clinic. Nephrol. Ther..

[4] Lv W., Booz G.W., Wang Y., Fan F., Roman R. (2018). Inflammation and renal fibrosis: Recent developments on key signaling molecules as potential therapeutic targets. Eur. J. Pharmacol..

[5] Zeisberg M., Neilson E.G. (2010). Mechanisms of tubulointerstitial fibrosis. J. Am. Soc. Nephrol..

[6] Gerarduzzi C., Di Battista J.A. (2017). Myofibroblast repair mechanisms post-inflammatory response: A fibrotic perspective. Inflamm. Res..

[7] Ghosh M., Tucker D., Burchett S., Leslie C. (2006). Properties of the Group IV phospholipase A2 family. Prog. Lipid Res..

[8] Khan N.S., Song C.Y., Thirunavukkarasu S., Fang X.R., Bonventre J.V., Malik K.U. (2016). Cytosolic Phospholipase A2alpha Is Essential for Renal Dysfunction and End-Organ Damage Associated with Angiotensin II-Induced Hypertension. Am. J. Hypertens..

[9] Montford J.R., Lehman A.M., Bauer C.D., Klawitter J., Klawitter J., Poczobutt J.M., Scobey M., Weiser-Evans M., Nemenoff R.A., Furgeson S.B. (2018). Bone marrow-derived cPLA2alpha contributes to renal fibrosis progression. J. Lipid Res..

[10] Ishihara K., Miyazaki A., Nabe T., Fushimi H., Iriyama N., Kanai S., Sato T., Uozumi N., Shimizu T., Akiba S. (2012). Group IVA phospholipase A2 participates in the progression of hepatic fibrosis. FASEB J..

[11] Furman B.L. (2015). Streptozotocin-Induced Diabetic Models in Mice and Rats. Curr. Protoc. Pharmacol..

[12] Holmeide A.K., Skattebol L. (2000). Syntheses of some polyunsaturated trifluoromethyl ketones as potential phospholipase A(2) inhibitors. J. Chem. Soc.-Perkin Trans..

[13] Bonventre J.V., Huang Z., Taheri M.R., O’LEary E., Li E., Moskowitz M.A., Sapirstein A. (1997). Reduced fertility and postischaemic brain injury in mice deficient in cytosolic phospholipase A2. Nature.

[14] Quehenberger O., Dahlberg-Wright S., Jiang J., Armando A.M., Dennis E.A. (2018). Quantitative determination of esterified eicosanoids and related oxygenated metabolites after base hydrolysis. J. Lipid Res..

[15] Lamprecht M.R., Sabatini D.M., Carpenter A.E. (2007). CellProfiler: Free, versatile software for automated biological image analysis. Biotechniques.

[16] Tollemar V., Tudzarovski N., Boberg E., Andrén A.T., Al-Adili A., Le Blanc K., Legert K.G., Bottai M., Warfvinge G., Sugars R. (2018). Quantitative chromogenic immunohistochemical image analysis in cellprofiler software. Cytom. A.

[17] Branton M.H., Kopp J.B. (1999). TGF-beta and fibrosis. Microbes Infect..

[18] Halper J., Kjaer M. (2014). Basic components of connective tissues and extracellular matrix: Elastin, fibrillin, fibulins, fibrinogen, fibronectin, laminin, tenascins and thrombospondins. Adv. Exp. Med. Biol..

[19] Feuerherm A.J., Dennis E.A., Johansen B. (2019). Cytosolic group IVA phospholipase A2 inhibitors, AVX001 and AVX002, ameliorate collagen-induced arthritis. Arthritis Res. Ther..

[20] Huang L., Scarpellini A., Funck M., Verderio E.A., Johnson T.S. (2013). Development of a chronic kidney disease model in C57BL/6 mice with relevance to human pathology. Nephron Extra.

[21] Reginster F., Jadoul M., van Ypersele de Strihou C. (1997). Chinese herbs nephropathy presentation, natural history and fate after transplantation. Nephrol. Dial. Transplant..

[22] Rui H.-L., Wang Y.-Y., Cheng H., Chen Y.-P. (2012). JNK-dependent AP-1 activation is required for aristolochic acid-induced TGF-beta1 synthesis in human renal proximal epithelial cells. Am. J. Physiol. Ren. Physiol..

[23] Chen S., Wang Y., Wan Y. (2017). Urotensin II enhances transforming growth factorbeta1 expression and secretion in the kidney during aristolochic acid nephropathy. Mol. Med. Rep..

[24] Tesch G.H., Allen T.J. (2007). Rodent models of streptozotocin-induced diabetic nephropathy. Nephrology.

[25] Hirose S., Yamamoto T., Feng L., Yaoita E., Kawasaki K., Goto S., Fujinaka H., Wilson C.B., Arakawa M., Kihara I. (1998). Expression and localization of cyclooxygenase isoforms and cytosolic phospholipase A2 in anti-Thy-1 glomerulonephritis. J. Am. Soc. Nephrol..

[26] Aukema H.M., Adolphe J., Mishra S., Jiang J., Cuozzo F.P., Ogborn M.R. (2003). Alterations in renal cytosolic phospholipase A2 and cyclooxygenases in polycystic kidney disease. FASEB J..

[27] Cybulsky A.V., Takano T., Papillon J., McTavish A.J. (2000). Complement-induced phospholipase A2 activation in experimental membranous nephropathy. Kidney Int..

[28] Furuya Y., Tagami S., Hasegawa A., Ishii J., Hirokawa J., Yoshimura H., Honda T., Sakaue S., Aoki K., Murakami M. (1999). Increased glomerular cytosolic phospholipase A2 activity of OLETF rats with early diabetes. Exp. Clin. Endocrinol. Diabetes.

[29] (2020). Nephroseq. https://available-inventions.umich.edu/product/nephroseq.

[30] Papadopoulos T., Krochmal M., Cisek K., Fernandes M., Husi H., Stevens R., Bascands J.-L., Schanstra J.P., Klein J. (2016). Omics databases on kidney disease: Where they can be found and how to benefit from them. Clin. Kidney J..

[31] Berthier C.C., Bethunaickan R., Gonzalez-Rivera T., Nair V., Ramanujam M., Zhang W., Bottinger E.P., Segerer S., Lindenmeyer M., Cohen C.D. (2012). Cross-species transcriptional network analysis defines shared inflammatory responses in murine and human lupus nephritis. J. Immunol..

[32] Ju W., Greene C.S., Eichinger F., Nair V., Hodgin J.B., Bitzer M., Lee Y.-S., Zhu Q., Kehata M., Li M. (2013). Defining cell-type specificity at the transcriptional level in human disease. Genome Res..

[33] Reich H.N., Tritchler D., Cattran D.C., Herzenberg A.M., Eichinger F., Boucherot A., Henger A., Berthier C.C., Nair V., Cohen C.D. (2010). A molecular signature of proteinuria in glomerulonephritis. PLoS ONE.

[34] Woroniecka K.I., Park A.S.D., Mohtat D., Thomas D.B., Pullman J.M., Susztak K. (2011). Transcriptome analysis of human diabetic kidney disease. Diabetes.

[35] Grotendorst G.R., Rahmanie H., Duncan M.R. (2004). Combinatorial signaling pathways determine fibroblast proliferation and myofibroblast differentiation. FASEB J..

[36] Huwiler A., Feuerherm A.J., Sakem B., Pastukhov O., Filipenko I., Nguyen T., Johansen B. (2012). The omega3-polyunsaturated fatty acid derivatives AVX001 and AVX002 directly inhibit cytosolic phospholipase A(2) and suppress PGE(2) formation in mesangial cells. Br. J. Pharmacol..

[37] Sommerfelt R.M., Feuerherm A.J., Jones K., Johansen B. (2013). Cytosolic phospholipase A2 regulates TNF-induced production of joint destructive effectors in synoviocytes. PLoS ONE.

[38] Sugimoto Y., Narumiya S. (2007). Prostaglandin E receptors. J. Biol. Chem..

[39] Sarnak M.J., Levey A.S., Schoolwerth A.C., Coresh J., Culleton B., Hamm L.L., McCullough P.A., Kasiske B.L., Kelepouris E., Klag M.J. (2003). Kidney Disease as a Risk Factor for Development of Cardiovascular Disease. Circulation.

[40] Meng X.-M., Nikolic-Paterson D.J., Lan H.Y. (2014). Inflammatory processes in renal fibrosis. Nat. Rev. Nephrol..

[41] Lee S.Y., Kim S.I., Choi M.E. (2015). Therapeutic targets for treating fibrotic kidney diseases. Transl. Res..

[42] Vega G., Alarcon S., San Martin R. (2016). The cellular and signalling alterations conducted by TGF-beta contributing to renal fibrosis. Cytokine.

[43] Hao C.M., Breyer M.D. (2007). Roles of lipid mediators in kidney injury. Semin Nephrol..

[44] Montford J.R., Bauer C., Dobrinskikh E., Hopp K., Levi M., Weiser-Evans M., Nemenoff R., Furgeson S.B. (2019). Inhibition of 5-Lipoxygenase Decreases Renal Fibrosis and Progression of Chronic Kidney Disease. Am. J. Physiol. Ren. Physiol..

[45] Singh N.K., Rao G.N. (2019). Emerging role of 12/15-Lipoxygenase (ALOX15) in human pathologies. Prog. Lipid Res..

