# Supplementary material for: Group IVA Cytosolic Phospholipase A2 (cPLA2α): Pharmacological Inhibition Attenuates Kidney Inflammation and Fibrosis
Source: Biomolecules. 2026 Oct 2;16(10):1430. doi: 10.3390/biom16101430 (PMC13702105; doi:10.3390/biom16101430)
Supplement: Supplementary file 1 [file biomolecules-16-01430-s001.zip › Figure S1.pdf]

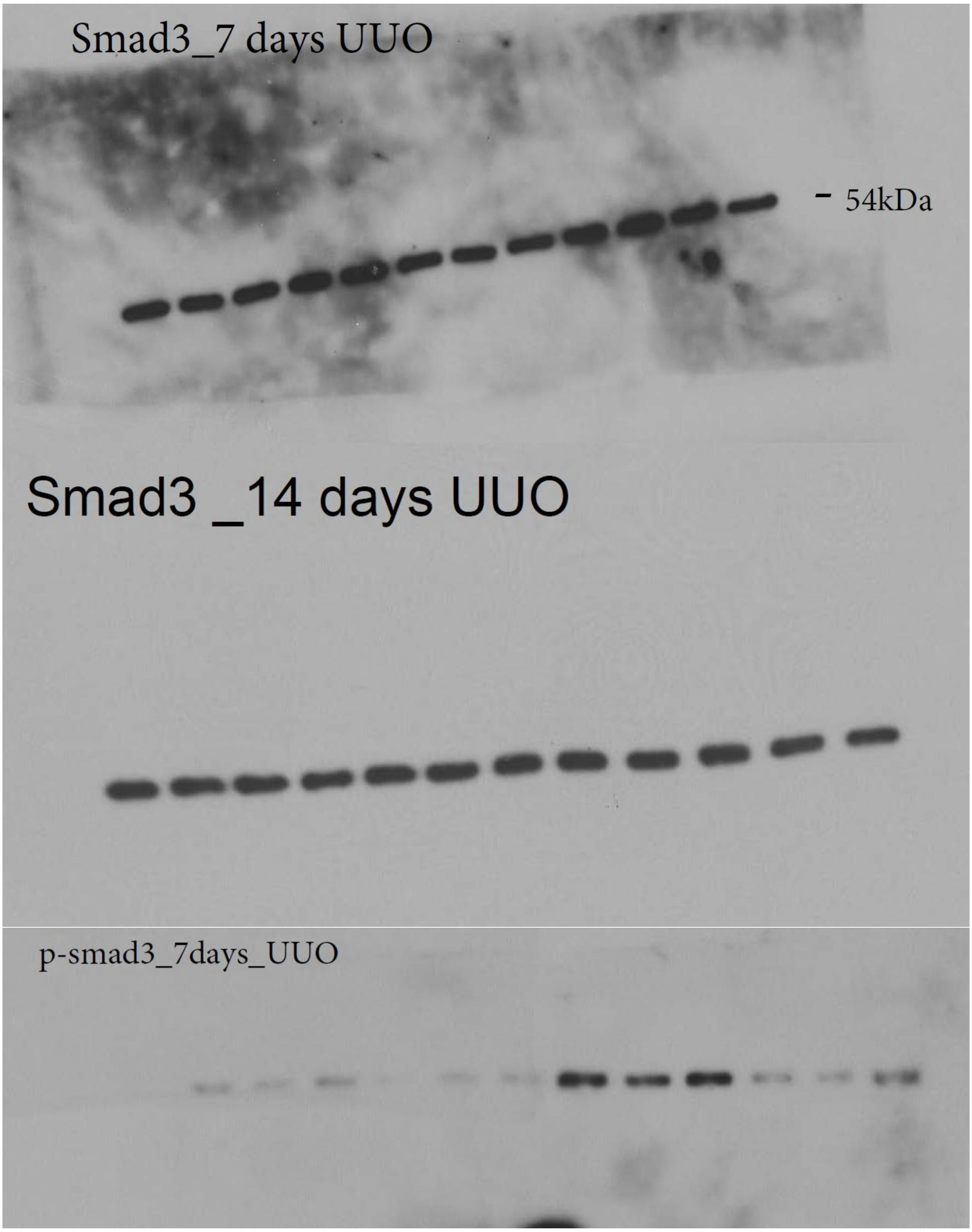
Smad3_7 days UUO
54kDa
Smad3 _14 days UUO
p-smad3_7days_UUO

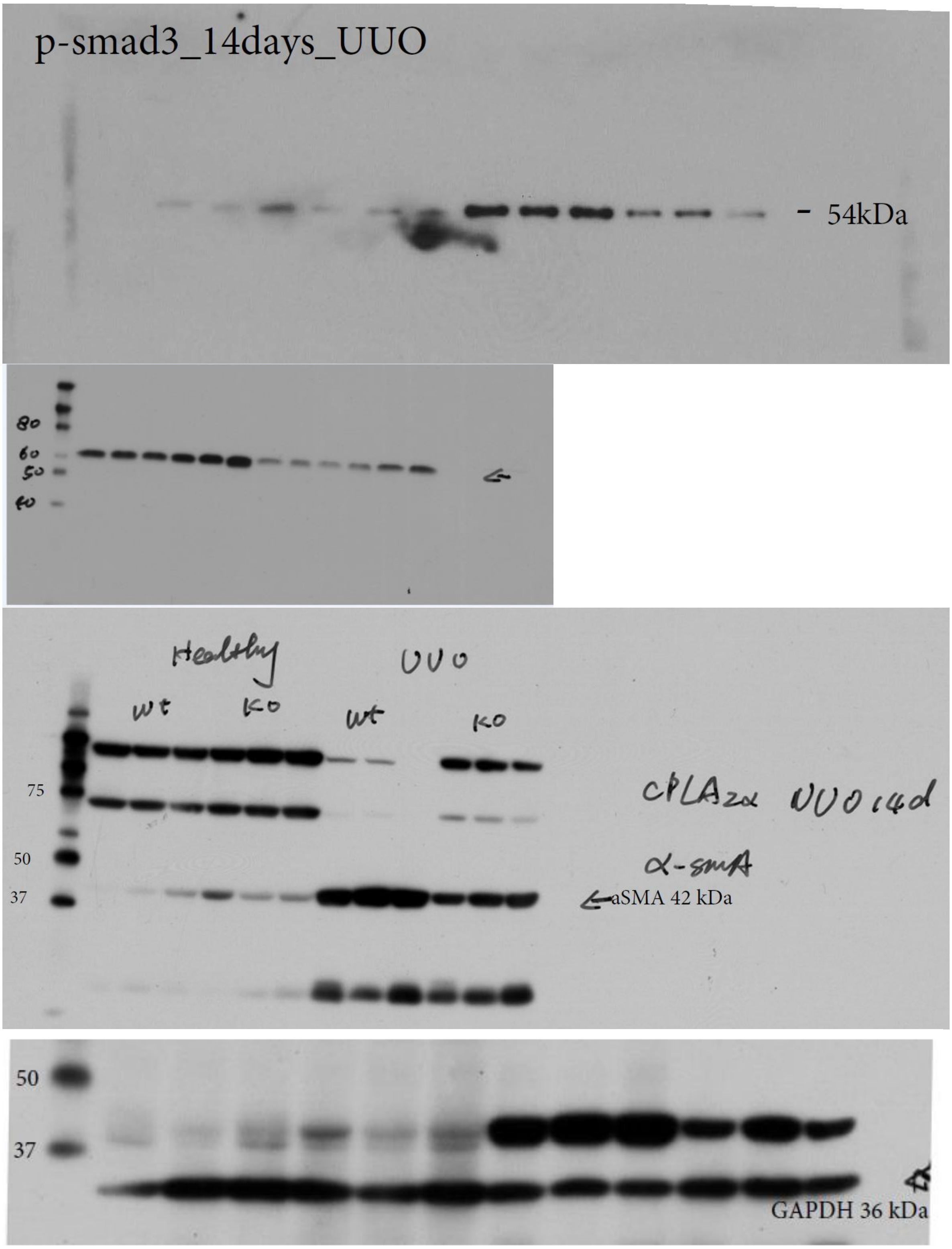
p-smad3_14days_UUO
54kDa
80
60
50
40
Healthy
UUO
wt
KO
wt
KO
75
50
37
cPLA2α UUO 14d
α-SMA
aSMA 42 kDa
50
37
GAPDH 36 kDa

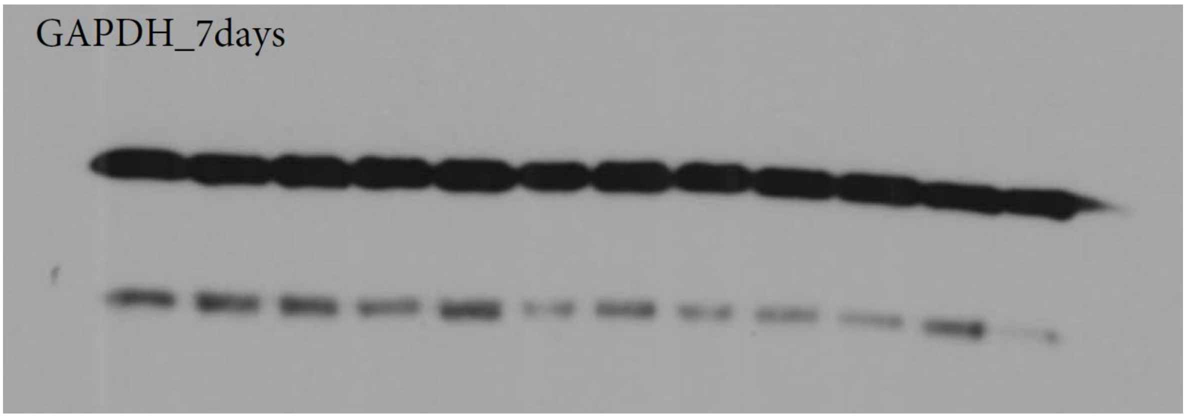
GAPDH_7days

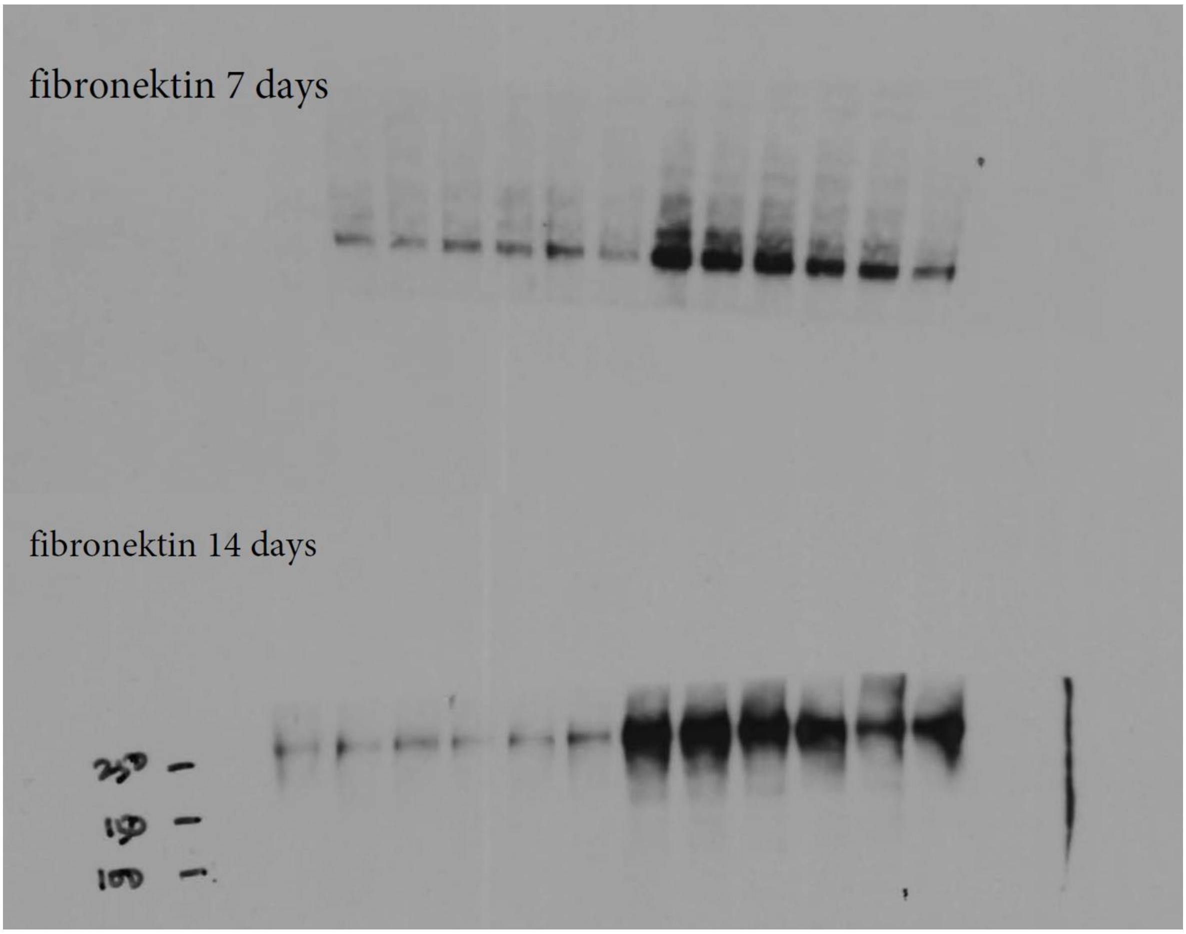
fibronektin 7 days
fibronektin 14 days
250
150
100
